# A Primer for Using Transgenic Insecticidal Cotton in Developing Countries

**DOI:** 10.1673/031.009.2201

**Published:** 2009-05-22

**Authors:** Ann M. Showalter, Shannon Heuberger, Bruce E. Tabashnik, Yves Carrière

**Affiliations:** Department of Entomology, University of Arizona, Tucson AZ

**Keywords:** Agronomic performance, *Bacillus thuringiensis*, gene flow, non-target effects, pest control, resistance management, transgene expression, transgenic cotton

## Abstract

Many developing countries face the decision of whether to approve the testing and commercial use of insecticidal transgenic cotton and the task of developing adequate regulations for its use. In this review, we outline concepts and provide information to assist farmers, regulators and scientists in making decisions concerning this technology. We address seven critical topics: 1) molecular and breeding techniques used for the development of transgenic cotton cultivars, 2) properties of transgenic cotton cultivars and their efficacy against major insect pests, 3) agronomic performance of transgenic cotton in developing countries, 4) factors affecting transgene expression, 5) impact of gene flow between transgenic and non-transgenic cotton, 6) non-target effects of transgenic cotton, and 7) management of pest resistance to transgenic cotton.

## Introduction

More than two-thirds of the world's cotton is produced by countries with developing economies. Cotton is an important cash crop in such countries as it supports the livelihoods of millions of households, may represent a significant part of a country's total export, or play a key role in sustaining textile industries that create employment opportunities (Baffes 2005). Worldwide intensification of cotton production and the evolution of resistance to synthetic insecticides in major cotton pests are key factors influencing the use of insecticidal transgenic cotton in the developing world (Baffes 2005, Wu and Guo 2005, Bennett et al. 2006, Vassayre et al. 2006).

Transgenic cotton was first used commercially in 1996 in Argentina, Australia, China, Mexico, and the United States (James 2006). In 2006, more than 13 million hectares of transgenic cotton producing toxins from the soil bacterium *Bacillus thuringiensis* (Bt) were cultivated in nine countries on five continents; more than 10 million resource-poor farmers used this technology in the six developing countries (Brazil, China, Colombia, India, Mexico, South Africa) where it is authorized (James 2006). While the use of transgenic cotton continues to increase, many developing countries face the decision of whether to approve the testing and commercial use of transgenic cotton and the task of developing adequate regulations for its use.

Much has been learned about the benefits and risks associated with transgenic crops with insecticidal properties. Here we outline concepts and provide information about transgenic cotton that will assist farmers, regulators and scientists in developing countries. We start with a general outline of the molecular and breeding techniques used to create transgenic cotton cultivars. We then describe properties of commercialized and near-commercialized transgenic cotton cultivars and review their efficacy against major cotton pests. Following this, we assess the agronomic performance of transgenic cotton across a representative set of developing countries. In a subsequent section, we focus on factors that affect transgene expression. In the last three sections, we examine risks associated with the deployment of transgenic cotton and factors that may affect its long-term performance: these factors include the impact of gene flow between transgenic and non-transgenic cotton, non-target effects, and the management of pest resistance. We conclude with a summary of key findings and research needs. A glossary of technical terms follows.

### Development of transgenic cotton cultivars

With the exception of steps involved in the insertion of foreign DNA into cotton, the development of transgenic cotton cultivars is similar to conventional cotton breeding. Understanding how transgenic cotton cultivars are developed can have important implications for policy and management decisions. Below, we describe the main techniques currently used to produce transgenic cotton cultivars.

### Insertion of foreign DNA into cotton

The process that makes transgenic cotton different from conventional cotton is the insertion of DNA from a different organism into the plant's genome. The inserted DNA, or transgenic DNA, generally consists of three main parts: a gene of interest, a promoter, and a marker gene. The gene of interest produces a novel characteristic (e.g., the production of an insecticidal protein from the bacterium *Bacillus thuringiensis*) that could not be developed through conventional plant breeding. Insertion of this gene alone would not reliably produce the desired characteristic without the promoter. A promoter is a regulatory sequence of DNA that determines where, when, and how much of a gene is expressed. Promoters can be constitutive, tissue-specific, or inducible. The final part of transgenic DNA is a marker gene, which produces a selectable characteristic (e.g., resistance to an antibiotic or herbicide). Expression of the marker gene signifies that the gene of interest has been successfully transferred to the plant's genome.

Many techniques for transforming plants are available, but the development of most commercial transgenic cotton cultivars has been conducted using one of three techniques: *Agrobacterium*-mediated transformation, particle bombardment, or the pollen-tube pathway (Potrykus et al. 1998, Somers et al. 2004, Xue et al. 2006). *Agrobacterium*-mediated transformation is the most widely used technique. This technique uses the natural ability of the plant pathogen *Agrobacterium tumefaciens* to transfer a plasmid into a plant's genome. To transform cotton, scientists create a plasmid containing the desired transgenic DNA (transgene with promoter and marker gene) that is absorbed by the bacterium. Plant tissue or cell cultures are inoculated with the *Agrobacterium*, which transfers the transgenic DNA to the plant's genome. Inoculated plant tissue in which the marker gene is expressed is selected and allowed to regenerate into a whole cotton plant.

The second transformation technique is called particle bombardment. Like *Agrobacterium*-mediated transformation, particle bombardment requires plant cell or tissue cultures. However, instead of using a bacterium to transfer DNA, this technique uses ballistics. DNA-coated micro-projectiles are inserted into plant cells at high velocities using an instrument called a “gene gun”. Once the transgenic DNA enters the cell, it is absorbed into the recipient plant's genome. Plant tissues expressing the marker gene are selected and grown into whole plants.

**Figure 1.**
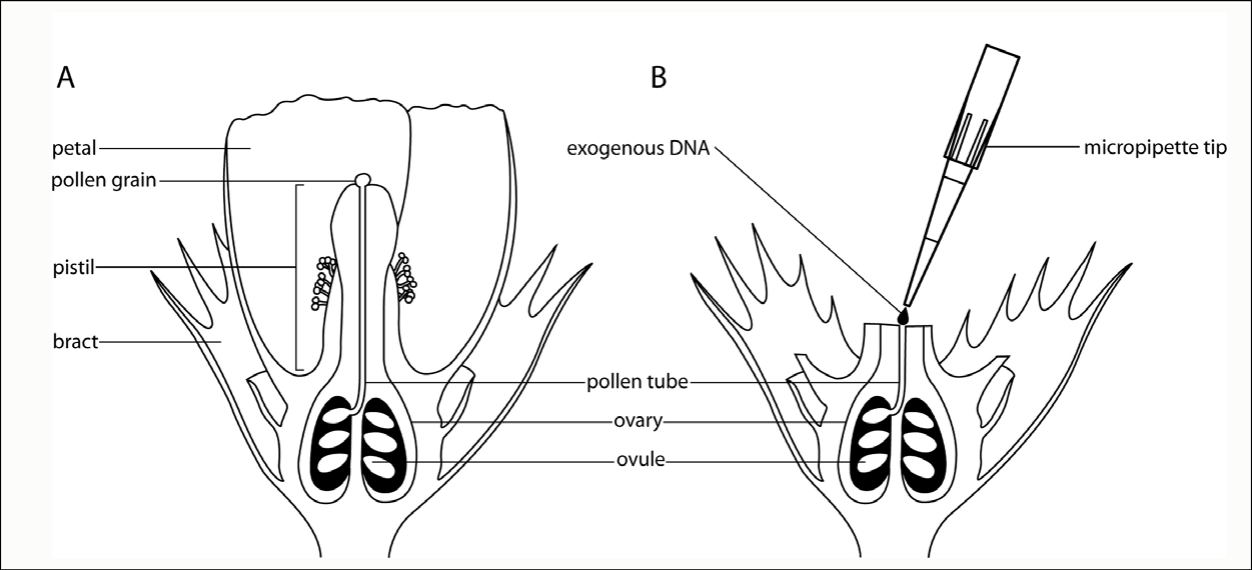
Pollen-tube pathway transformation technique. (A) Cross-section of a fertilized cotton flower (not to scale) with a pollen tube connecting the pollen grain and an ovule. (B) The petals and top of the pistil are removed from the fertilized flower to expose the ovary. Exogenous DNA containing a transgene(s) is injected into the exposed ovary, where the DNA travels down the pollen tube to the fertilized ovule.

The final technique, the pollen-tube pathway, is distinct from the previous methods because it does not require cell or tissue cultures. Flowering cotton plants are allowed to self-pollinate. The plant produces a pollen tube from the tip of the pistil to the ovule (Figure 1). Sperm produced by the pollen grain travels down the pollen tube to an ovule where the egg is located. Once this has occurred, the ovary, which contains the ovules, is exposed by removing the petals, and a solution containing transgenic DNA is injected into the ovary. The DNA travels down the pollen tube to the ovule and is absorbed into the genome of the developing cotton embryo (Zhou et al. 1983). When the ovules mature into seeds, the seeds are planted and selected for successful transgene integration.

Each transformation technique has advantages and disadvantages. *Agrobacterium*-mediated transformation and particle bombardment are both established and accepted techniques that have been used successfully to create many transgenic cotton cultivars. These techniques have consistently produced transgenic plants in which the transgene is expressed in subsequent generations. The largest drawback to these techniques is the required use of cell or tissue cultures for transformation. Most cotton cultivars cannot regenerate from these cultures. Thus, most scientists choose the American “Coker” cultivar as the recipient plant because it readily regenerates (Smith et al. 2004). In contrast, any cotton cultivar can be transformed using the pollen-tube pathway because regeneration is not required. In addition, the pollen-tube pathway does not require a marker gene for the selection process because other methods (e.g., PCR, Southern blot analysis) can efficiently determine whether a plant is transgenic. These methods are not feasible with *Agrobacterium*-mediated transformation or particle bombardment because of the sheer number of plants that would need to be analyzed (Potrykus et al. 1998, Somers et al. 2004). A cell or tissue culture can contain tens of millions of cells, and only a fraction of these cells will be successfully transformed (Potrykus et al. 1998, Somers et al. 2004). Rearing all these cells into whole plants that can be analyzed with PCR and Southern blot analyses would require an extraordinary amount of resources. Although the pollentube pathway has been used in Chinese biotechnology (Pray and Huang 2003, Xue et al. 2006), this technique remains controversial because results are often inconsistent or irreproducible (Twyman et al. 2002, Xue et al. 2006). For example, some plants may appear to be transformed because they express the desired transgene, but the transgenic characteristic may not persist in subsequent generations (Zhang et al. 2005a). To date, no studies comparing transformation techniques in cotton have been published, although results in maize suggest that *Agrobacterium*-mediated transformation is marginally more successful than the other two methods (Zhang et al. 2005).

### Development of commercial cultivars and hybrids

After whole transgenic cotton plants have been regenerated from tissue (*Agrobacterium*-mediated or particle bombardment) or grown from seed (pollen-tube pathway), a rigorous selection process is undertaken to identify plants with good agronomic characteristics and with the highest and most consistent levels of transgene expression. The selection process begins by eliminating transformed plants with obvious abnormalities. Abnormalities can occur from disruptive mutations that may arise during the tissue culture and regeneration process (Sachs et al. 1998). Moreover, because the site of transgene insertion (locus) in the plant's genome is random, abnormalities in plant architecture or physiology can arise when a transgene is inserted in an important plant gene or promoter and disrupts the expression of plant genes (Sachs et al. 1998). Plants with normal development are then analyzed to determine the number of transgene loci and the level of transgene expression. Multiple transgene loci can reduce transgene expression, a phenomenon called gene silencing (Finnegan and McElroy 1994, Matzke and Matzke 1995). Thus, the ideal transgenic plant contains a single transgene locus that is highly expressed.

After eliminating abnormal and multi-loci individuals, as well as those with low transgene expression, the remaining transgenic plants are typically allowed to self-fertilize for a few generations to ensure that inheritance of the transgene is predictable and transgene expression remains stable (Skinner et al. 2004). If the transgene was improperly incorporated into the plant's genome, the transgene will not be reliably transmitted to the next generation (Zhang et al. 2005). During this stage, scientists may select individuals with particularly good agronomic characteristics. Another goal of the self-fertilization process is to produce cotton plants that are homozygous for the transgene locus. Homozygosity is important because all of the plant's progeny will contain a copy of the transgene even if the other parent is not transgenic. Homozygous plants are sometimes called “true-breeding”. The end result of this selection process is a true-breeding transgenic cultivar.

In cotton plants transformed using the *Agrobacterium*-mediated or ballistic technique, the true-breeding cultivar is rarely commercially useful, due to its Coker genetic background (Smith et al. 2004). To develop a commercial transgenic cultivar and eliminate the Coker genetic background, a series of backcrosses are conducted. This begins when the transgenic Coker line is crossed with an established commercial cultivar. The hybrid progeny express the transgene and agronomic characteristics of the commercial cultivar. Because the hybrid also contains Coker genes that may negatively affect performance, the hybrids are backcrossed with the commercial parent cultivar. Backcrossing dilutes the proportion of Coker genes that comprise the plant's genome, so the genetic background of the backcrossed progeny contains fewer Coker genes than that of the hybrids. For example, ignoring genes linked to the transgene (i.e., genes on the same chromosome as the insertion site), ca. 93%, 96%, 98%, and 99% of the Coker genes are replaced by genes of the commercial cultivar after 3, 4, 5, and 6 generations of backcrossing, respectively. With each generation, scientists carefully screen transgenic individuals for agronomic performance and relatedness to the commercial cultivar, and only individuals that perform well are used in the next backcross. Thus, backcrossing for 5ndash;10 generations can essentially eliminate Coker genes that affect performance and produce a transgenic cultivar that is nearly identical to the original commercial cultivar (Duck and Evola 1997). Hence, there is little reason to believe that the use of Coker lines for transformation purposes has any negative effect on the performance or genetic diversity of commercial transgenic cultivars. The last step is to self-fertilise plants that bear the transgene and retain progeny homozygous for the transgene. This yields a true-breeding commercial transgenic cultivar, which is important because all of the progeny produced by a homozygous transgenic individual will contain a copy of the transgene even if the other parent is not transgenic.

Because developing new true-breeding commercial cultivars using the backcrossing process is time-consuming, scientists may opt to produce hybrid cotton plants that can be developed in a single generation. Transgenic hybrids are developed by crossing a true-breeding transgenic cultivar (the homozygous product of the intense backcrossing process) and a non-transgenic cultivar with desirable agronomic characteristics. This cross produces hybrid seeds that contain characteristics of both varieties (e.g., the transgene and the desired agronomic characteristics), which can then be sold commercially. Hybridization between cotton cultivars has, until recently, been somewhat difficult to perform. Because cotton plants typically self-fertilize, breeders must hand-pollinate cotton plants to produce hybrids, a labor-intensive and potentially uneconomical process. Recent advances in breeding (e.g., inducing male sterility and fertility) have greatly improved the efficiency of this process (Zhang et al. 2000a, Dong et al. 2004). Although the exact techniques used to make a hybrid may vary, the end product (i.e., the hybrid seed) is the same.

Hybridization can result in plants that are more vigorous than either of their parental cultivars, a phenomenon called heterosis. However, not all transgenic hybrids perform better than their parental cultivars. For example, if one parent cultivar requires regular irrigation and the other parent is drought-tolerant, the hybrid progeny may be more sensitive to drought than the drought-tolerant parent. For this reason, cotton breeders must choose the most appropriate cultivars when developing hybrids and field test these hybrids to avoid any negative effects of parental genetic background.

### Efficacy of insecticidal transgenic cotton against arthropod pests

During the first years of transgenic cotton use, most transgenic cotton cultivars produced the *Bacillus thunrigiensis* (Bt) toxin Cry1Ac or Cry1A (Table 1). However, the number of transgenes available in cotton has expanded, and several transgenic cultivars and hybrids are currently available worldwide. Each transgene offers protection from some of the most economically important cotton pests.

### Types of insecticidal transgenes

Many proteins have been investigated for their insecticidal properties, but only nine are commercially available or may soon become available in transgenic cotton cultivars (Table 1). These genes and the toxins they produce can be grouped into four categories: Bt crystalline δ-endotoxins, Bt vegetative insecticidal proteins (Vip), proteinase inhibitors, and lectins.

Crystalline (Cry) δ-endotoxins from the soil bacterium *Bacillus thuringiensis* have been the most extensively studied and used in transgenics. Once activated by insect proteases in the insect midgut, Cry proteins bind to receptors in the midgut (Schnepf et al. 1998). Such binding leads to the formation of pores in the midgut membrane and ultimately to cell lysis and death. The specific binding of Cry toxins to midgut membrane receptors is a key determinant of pest specificity (Schnepf et al. 1998). Five *cry* genes (*cry1A, cry1Ac, cry2Ab, cry1F*, and *cry1EC*) are commercially available or nearly commercialized in cotton (Table 1). While *cry1A* designates a family of genes, *cry1A* is also used to describe Chinese cultivars that contain a fusion of *cry1Ac* and *cry1Ab* genes (Huang et al. 2002b, Pray and Huang 2003, Dong et al. 2004).

**Table 1.**
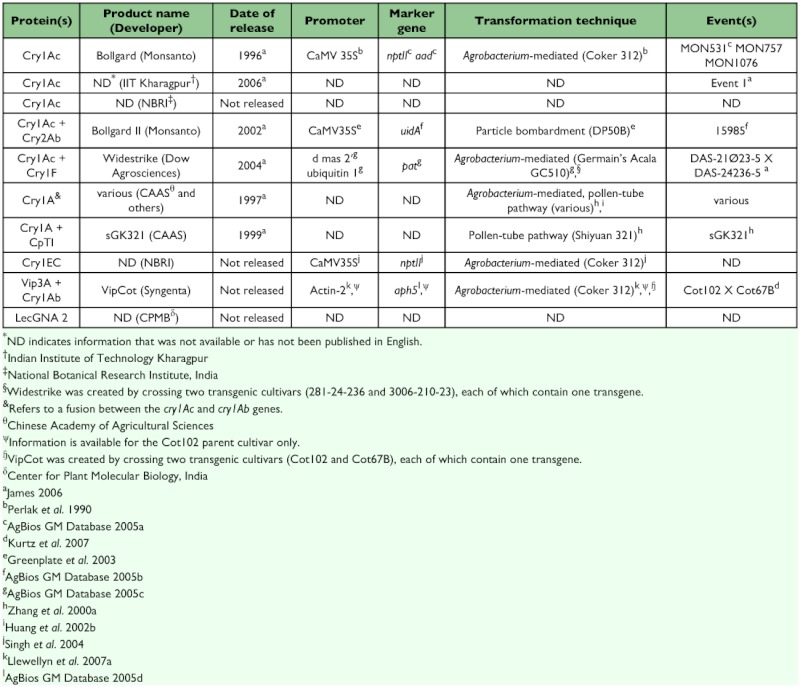
Characteristics of commercialized or near-term transgenic cotton cultivars.

The second group of toxins, vegetative insecticidal proteins (Vips), is also derived from *Bacillus thuringiensis*. Vip toxins affect insects in a manner similar to that of Cry proteins. However, Vips bind to different receptors on midgut cells (Lee et al. 2003). None of the cultivars with Vip toxins are commercially available to date (Table 1).

The third group of toxins used in cotton transgenics includes protease inhibitors, which are typically derived from plant proteins. Protease inhibitors inactivate the enzymes in an insect's gut that digest proteins. This leads to amino acid deficiencies that cause delayed development and death (Hilder et al. 1989). The cowpea trypsin inhibitor (CpT1) is one protease inhibitor that has been successfully introduced into cotton and is available commercially (Table 1).

The final group of toxins currently used in cotton transgenics is lectins. Lectins are proteins that bind to carbohydrates. Like protease inhibitors, they are found in many plant species. The exact mode of action of these toxins remains uncertain, although lectins are known to bind to the carbohydrate receptors on insect midgut cells and negatively affect gut function and iron metabolism (Tinjuangjun 2002). The use of the snowdrop lectin gene (*lecGNA 2*) in cotton is being investigated in India but has not been commercially released (Table 1; Jayaraman 2004, James 2006).

### Efficacy of transgenic cotton

There are more than 1300 arthropod pests of cotton around the world (Matthews and Tunstall 1994). We focus on the 12 most economically important of these pests, as efficacy data is available for many of them, particularly the ones found in the United States, India, and China. Information on the efficacy of transgenic cotton is much more extensive in older cultivars (e.g., Bollgard) than in recently developed ones. Although efficacy can be quantified in several ways, we focused on studies that measured mortality using bioassays (Table 2, Appendix A) and studies that compared densities of insect populations between transgenic and non-transgenic plots or fields (Table 3, Appendix B). Bioassays are more easily conducted than field experiments and provide controlled estimates of efficacy. However, field studies of pest densities may be more representative of the control farmers can expect from transgenic cotton cultivars.

Most transgenic cultivars target lepidopteran pests, which include bollworms, *Helicoverpa* spp.; tobacco budworm, *Heliothis virescens*; pink bollworm, *Pectinophora gossypiella*; spiny/spotted bollworms, *Earias* spp.; and red bollworms, *Diparopsis* spp. (Table 2 and 3). In many nations, these species are the most economically important cotton pests (Hearn and Fitt 1992, Matthews and Tunstall 1994). Most of these bollworms and budworms are moderately to highly susceptible to the Cry toxins found in Bollgard, Bollgard II, Widestrike, and Chinese Cry1A cultivars (Table 2 and 3). However, Cry toxins are generally less effective against *Helicoverpa* spp. than the other insects in this group. Cotton with the Vip toxin (VipCot) provides moderate to high levels of protection against bollworms and budworms, while the recently developed LecGNA 2 cotton cultivar, which targets pests other than bollworms and budworms (see below), only offers low protection against *Helicoverpa* spp. Several bollworm species in the genera *Earias* and *Diparopsis*, which are important pests in parts of Asia and Africa, have not been tested for their susceptibility to most transgenic cultivars.

Armyworms (*Spodoptera* spp.) are close relatives of bollworms and can be important pests of cotton in certain parts of the world (Hearn and Fitt 1992, Matthews and Tunstall 1994). They are poorly to moderately controlled by the toxins found in Bollgard and Chinese Cry1A cultivars (Table 2 and 3). However, newer transgenic cultivars such as Bollgard II, Widestrike, and VipCot were designed to confer greater resistance against armyworms than older varieties such as Bollgard. Bollgard II and Widestrike offer low to high levels of control of these pests. The range of control likely results from variation between studies and methodologies (see Appendix A and B). Cotton plants producing Cry1EC, a synthetic hybrid between Cry1E and Cry1C, were developed specifically to target armyworms. Cry1EC cotton killed 100% of all tested *S. litura* life stages (Table 2). Currently, no data are available on the efficacy of CpTI or LecGNA 2 against armyworms.

The remaining important cotton pests include the jassids, leafhoppers, aphids, mirids, whiteflies, thrips, and mites. The importance of these pests in cotton agriculture varies regionally (Hearn and Fitt 1992, Matthews and Tunstall 1994). Because Bt toxins are specific and most transgenic cotton has been engineered to target Lepidoptera, these cotton pests are unaffected by Bollgard, Widestrike, and VipCot (Table 2 and 3). Indeed, studies of the efficacy of transgenic cultivars are rarely published for these pests. However, LecGNA 2 that produces lectins targets aphids (Table 1).

### Performance of transgenic cotton in developing countries

The majority of published studies on transgenic cotton performance have documented positive results in developed and developing countries. Both large and small farmers using insecticidal transgenic cotton usually increased yields, decreased insecticide use, or both compared to non-users (Carpenter and Gianessi 2003, Fernandez-Cornejo et al. 2003, Fitt 2003, Kalaitzandonakes and Suntornpithug 2003, Qaim and de Janvry 2003, Traxler et al. 2003, Huesing and English 2004, Cattaneo et al. 2006, Raney 2006). The reduced costs of insecticide applications and increased yield resulted in economic gains for many small farmers, despite higher seed costs for transgenic cotton (Qaim and de Janvry 2003, Traxler et al. 2003, Huesing and English 2004, Hillocks 2005, Raney 2006). The following sub-sections summarize the results of studies on the performance of transgenic cotton in three developing countries for which there is relatively abundant data: China, India, and South Africa. These countries were selected because they provide contrasting insights into the benefits and concerns about the performance of transgenic cotton. Worldwide use of transgenic cotton was 13.4 million hectares in 2006 (James 2006). In 2006, China and India respectively used 3.5 and 3.8 million hectares of insecticidal transgenic cotton, while South Africa used 22,000 hectares of insecticidal and herbicide tolerant cotton.

### China

China has recently become the eading cotton-producing nation in the world (Hillocks 2005, Wu and Guo 2005). Soil and climatic conditions are favorable for cotton growing, and pest pressure is moderate with approximately 15% yield losses due to insect damage (Qaim and Zilberman 2003). Although large-scale cotton production does occur in China, the vast majority of cotton producers have farms of 0.4–2 hectares (Pray et al. 2001, Huang et al. 2002a, Huesing and English 2004, Hillocks 2005). As a result of intense government involvement in agriculture, cotton growers have access to subsidized insecticides and extension services (Pray et al. 2001, Huang et al. 2002b, Qaim and Zilberman 2003, Hillocks 2005, Wu and Guo 2005). Public development of transgenic cultivars has provided growers with several options of transgenic cultivars and hybrids (see below). In addition, transgenic cottonseed costs are not much higher than the costs of conventional cottonseed (Pray et al. 2001, Huang et al. 2002b, Huesing and English 2004, Raney 2006). This is largely a result of the availability of black market transgenic seeds (Huesing and English 2004), a practice that could reduce incentives for continued development of transgenic cultivars (Pray et al. 2001).

**Table 2.**
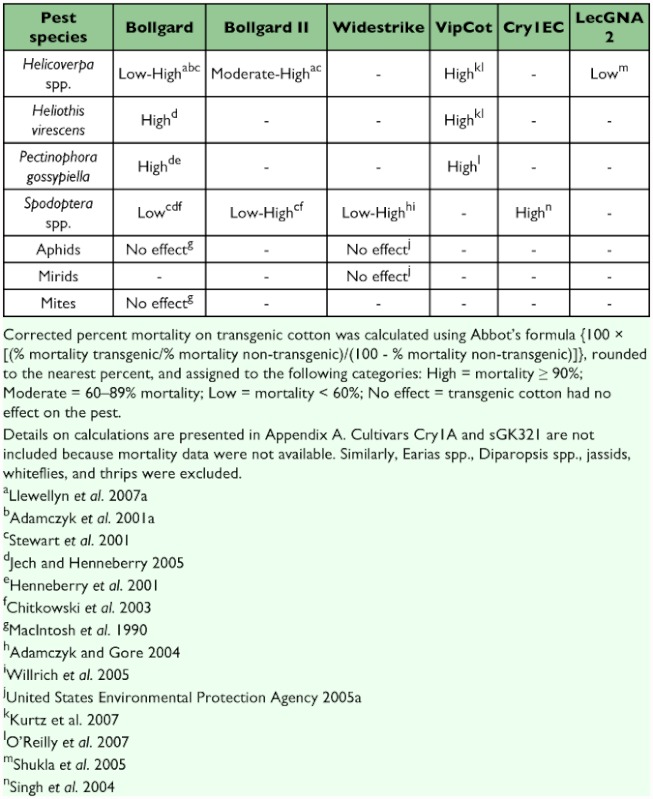
Efficacy of transgenic cotton cultivars against key cotton pests based on corrected percent mortality.

Easy access to inexpensive insecticides led to increased insecticide resistance in bollworms (primarily *Helicoverpa amigera*) during the 1990s (Zhang et al. 2000a, Huang et al. 2002b, Huesing and English 2004, Hillocks 2005). As a result, scientists at the Chinese Academy of Agricultural Sciences (CAAS) began developing transgenic cotton cultivars to control these pests (Pray et al. 2001, Xue et al. 2006). The first transgenic varieties developed by CAAS contained the Bt gene *cry1A* (Table 1). In 1997, four cultivars with *cry1A* were approved for commercialization (Pray et al. 2001, Dong et al. 2004, James 2006). A year later, Monsanto's American cultivar containing *cry1Ac* was released commercially in China (Pray et al. 2001, Huang et al. 2002b, Dong et al. 2004). In 1999, CAAS scientists developed another transgenic cultivar containing genes for Cry1A and the cowpea trypsin inhibitor CpTI (Table 1; Zhang et al. 2000a, James 2006). In the same year, techniques for efficiently hybridizing cotton by inducing male sterility and fertility were developed by Chinese researchers (Dong et al. 2004). As a result of these innovations, Chinese cotton growers have a relatively large selection of transgenic cultivars and hybrids available for planting. Nationally, the transgenic cotton adoption was about 66% of cotton planted in 2006 James 2006), with some areas reaching 100% (Li et al. 2007).

In general, gains in yield for Chinese adopters of transgenic cotton have been modest. Some studies demonstrated yield gains of 8–15% for transgenic cultivars over non-isogenic conventional cultivars (Huang et al. 2002b), whereas others showed that yields for transgenic cotton were approximately the same or slightly lower (e.g., reduced by 1.3%) than for non-isogenic conventional cotton (Zhang et al. 2000a, Pray and Huang 2003, Dong et al. 2004). Surveys of cotton growers indicated that resource-poor, small farmers obtained bigger yield gains and profits from adopting transgenic cotton than wealthier, larger farmers (Pray et al. 2001, Pray and Huang 2003). Performance studies also demonstrated 10–20% yield increases for hybrid transgenic cotton compared to purebred transgenic cultivars or hybrid and non-hybrid conventional cultivars (Zhang et al. 2000a, Dong et al. 2004).

**Table 3.**
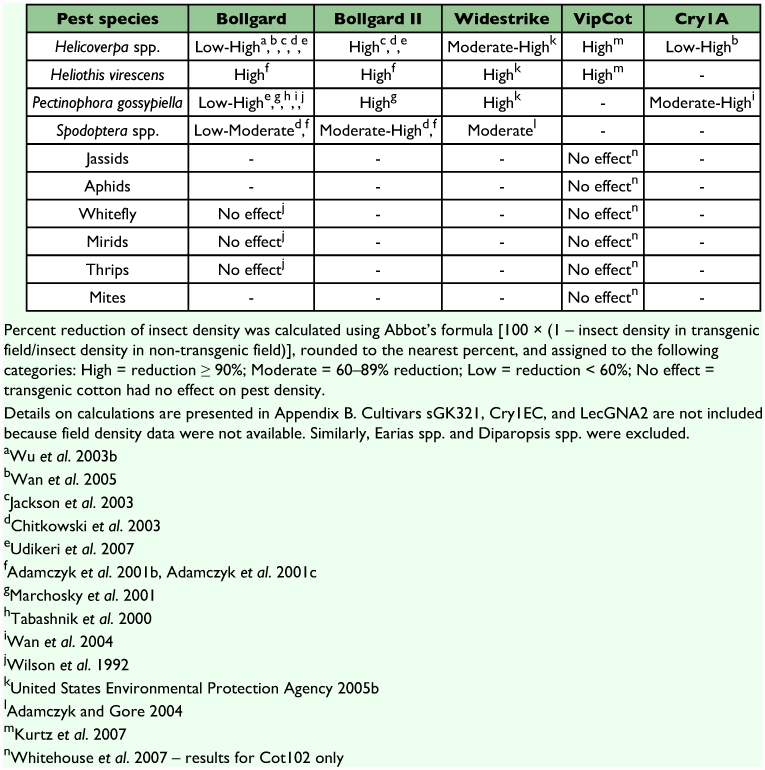
Efficacy of transgenic cotton cultivars against key cotton pests based on reduction of field pest density in transgenic fields compared to nontransgenic fields.

Variation between studies can be attributed to differences in the intensity of pest infestations between regions and years, as well as the performance of the conventional cultivars used as controls (Pray and Huang 2003). Because studies of cotton performance in China did not control for genetic background when comparing transgenic and conventional cultivars, the difference in performance may be a result of differences in genetic background rather than the presence of a transgene. The yield of a cultivar can be affected by many aspects of its genetic background such as drought-tolerance, tolerance to low soil fertility, resistance to pests and diseases, and fiber quality. Therefore, a comparison of isogenic transgenic and conventional varieties, which controls for the genetic background of a cultivar, would elucidate the benefits provided solely by the transgene(s). Despite this, the lack of large yield gains in China was expected because key cotton pests have been relatively well controlled by insecticides (Qaim and Zilberman 2003). Before the introduction of commercial transgenic cultivars, insect pests were adequately controlled in China despite increasing resistance to insecticides. Thus, the increased protection provided by transgenic cultivars did not result in large yield gains.

The primary benefit of transgenic cotton over non-isogenic conventional cotton in China has been reduced insecticide use. As a result of greater protection against bollworm damage with transgenic cotton, 47–75% less kilograms of insecticide, or 4–13 fewer insecticide applications, were needed to control these pests (Zhang et al. 2000a, Huang et al. 2002b, Dong et al. 2004). This reduced production costs and increased profits for large and smallholder adopters, even in cases where transgenic and non-transgenic cotton had similar yields (Zhang et al. 2000a, Huang et al. 2002b, Pray and Huang 2003). Reductions in insecticide use associated with transgenic cotton have varied regionally (Huang et al. 2002b). In regions where bollworms are not the primary cotton pests, farmers growing transgenic cotton observed a decrease in their insecticide use as low as 14% (Huang et al. 2002b). Therefore, farmers in regions with few bollworms may not benefit as much from adopting transgenic cotton as farmers in areas where bollworms are key pests. Moreover, transgene expression levels in Chinese cultivars generally decline over the growing season, which can reduce the efficacy of transgenic cultivars against bollworms (Wan et al. 2005). Thus, the use of transgenic cotton did not completely eliminate the need to spray for bollworms.

The introduction of transgenic cotton in China has had mixed effects on non-bollworm cotton pests. Cotton pests not affected by transgenic toxins (Table 2 and 3) still need to be controlled with synthetic insecticides. For example, acaricides (i.e., pesticides that target mites) are needed to sustain yields in both transgenic and non-transgenic cotton (Ma et al. 2006). Moreover, the use of transgenic cotton, which reduced insecticide use against bollworms, sometimes increased problems caused by other pests normally controlled by these insecticides. For example, reduction in the use of synthetic insecticides in transgenic cotton favored outbreaks of mirids and leafhoppers (Wu et al. 2002, Men et al. 2005). Such outbreaks likely reduced the yield benefits conferred by transgenic cotton. However, the use of transgenic cotton had mixed effects on non-bollworm cotton pests, as transgenic cotton provided indirect control of the aphid *Aphis gossypii*, an important cotton pest in parts of China. Insecticides sprayed to control bollworms decreased the number of natural enemies (predators and parasitoids) that kill aphids (Wu and Guo 2003). Because transgenic cotton reduced insecticidal sprays, aphid natural enemies remained abundant enough to control aphid populations, which likely increased the positive effects of transgenic cotton on yield (Wu and Guo 2003).

### India

Like China, India is a large producer of cotton (Huesing and English 2004, Bennett et al. 2005, Bennett et al. 2006). However, yields are relatively low compared to other countries due to a number of environmental factors and larger, more diverse insect pest populations (Qaim 2003. Qaim and Zilberman 2003, Huesing and English 2004. Bennett et al. 2005, Bennett et al. 2006). Soils can be less fertile than in other countries, and fertilizers that improve soil quality are infrequently applied (Qaim 2003, Huesing and English 2004). In addition, although rainfall patterns and other climatic conditions are often not optimal for cotton production (Qaim 2003, Huesing and English 2004), less than 40% of cotton fields are irrigated (Jayaraman 2002, Qaim 2003). Pest pressure, especially from bollworms (*Helicoverpa armigera* and *Earias* spp.), can cause yield losses of 50–60% (Qaim 2003, Qaim and Zilberman 2003, Huesing and English 2004, Bennett et al. 2005, Bennett et al. 2006). Insecticide use is common, although not as intense as in other countries (Qaim 2003, Qaim and Zilberman 2003, Hillocks 2005, Bennett et al. 2005). In addition, limited cash flow may restrict the growers' ability to purchase enough insecticides to be sprayed when they would be most effective, or the quality of insecticides may not always be adequate (Qaim 2003, Qaim and Zilberman 2003, Hillocks 2005, Bennett et al. 2005). With the exception of some largescale growers in the northern part of the country, most Indian cotton growers cultivate an average of 2–4 hectares (Qaim 2003, Huesing and English 2004, Qaim et al. 2006). Transgenic cottonseed can be 3–4 times more expensive than conventional cottonseed (Jayaraman 2002, Qaim 2003, Qaim et al. 2006). The high cost of transgenic seed has encouraged a black market in Bt seeds (Jayaraman 2002, Bennett et al. 2005, Qaim et al. 2006).

As in China, *H. armigera* has become increasingly resistant to insecticides in India (Sharma and Pampapathy 2006), and transgenic Bt cotton hybrids were developed to control *H. armigera* and other bollworms. In 2002, Maharashtra Hybrid Seeds Company commercially introduced cotton hybrids containing Monsanto's *cry1Ac* gene (Bennett et al. 2005, Tuli and Bhatia 2005, Qaim et al. 2006). The hybrids were developed by first producing a transgenic Indian cultivar via backcrossing and then hybridizing this transgenic cultivar with another local variety. For various political and economic reasons, only a limited number of Bt hybrids were approved for commercial release. In 2002 and 2003, three Bt hybrids were available to cotton growers (Qaim 2003, Tuli and Bhatia 2005, Qaim et al. 2006). By 2006, the number of official transgenic hybrids increased to 62, although illegal, unauthorized hybrids were also available in many states James 2006, Qaim et al. 2006). Among the official hybrids, hybrids containing *cry1Ac* and *cry2Ab* (Bollgard II, Monsanto) and *cry1A* (CAAS, China) have also become available to growers (James 2006). In 2006, transgenic hybrid cotton comprised 42% of the cotton planted in India James 2006).

In an effort to reduce their reliance on American transgenic germplasm, the Indian government recently invested in biotechnology research to develop its own transgenic cotton varieties. Scientists from the National Botanical Research Institute (NBRI), the Center for Plant Molecular Biology (CPMB), and the Indian Institute of Technology Kharagpur (IIT Kharagpur) succeeded in developing four different transgenic cotton cultivars. Two cultivars contain *cry1Ac* and the other two contain either *cry1EC* or the snowdrop lectin gene *lecGNA 2* (Jayaraman 2004, James 2006). The NBRI-developed *cry1Ac* gene is the same as Monsanto's, although NBRI researchers have modified the promoter, which they claim increased transgene expression levels Jayaraman 2004). In 2006, cotton containing the *cry1Ac* gene developed by IIT Kharagpur was commercially released James 2006). However, as of this writing, published information on transgenic cotton performance in India is mainly available for Bollgard hybrids (Table 2).

The biggest benefit of Bt hybrids for smallholder cotton growers in India is larger yields (Qaim 2003, Qaim and Zilberman 2003, Bennett et al. 2006). Field trials in 2001 showed a yield increase of 30–80% for transgenic hybrids over their conventional hybrid counterparts (Qaim 2003). Surveys in 2002 and 2003 showed that smallholder farmers growing Bt cotton in several regions had significantly higher yields (34–63%) than those who grew conventional cotton (Bennett et al. 2006, Qaim et al. 2006). Before the introduction of Bt cotton in India, many farmers were often unable to adequately control bollworms despite the use of insecticides (Qaim and Zilberman 2003). Because Bt hybrids experience one-third the boll loss due to insect damage relative to their non-Bt counterparts (Hebbar et al. 2007a), Bt cotton improved yields for farmers by providing increase protection against insects.

Although higher yields are the most important benefit of Bt cotton to Indian smallholder farmers, Bt adopters also used significantly less insecticide than farmers growing their non-Bt counterparts or other conventional varieties (Qaim 2003, Qaim and Zilberman 2003, Bennett et al. 2005, Bennett et al. 2006, Qaim et al. 2006). Studies from 2001 to 2003 found that 2.6–3 more insecticidal sprays for bollworms were used in conventional cotton fields than in Bt cotton fields (Qaim 2003, Qaim and Zilberman 2003, Qaim et al. 2006). Fewer sprays allowed smallholder farmers to reduce the amount of insecticide used on cotton by 33–50% (Qaim 2003, Qaim et al. 2006). Moreover, because many of the insecticides that target bollworms are highly toxic, Bt-adopting farmers significantly reduced their use of the most toxic insecticides (Qaim 2003, Qaim and Zilberman 2003). The overall reduction in insecticide use reduced production costs for farmers growing Bt cotton (Qaim 2003, Qaim and Zilberman 2003, Bennett et al. 2005, Qaim et al. 2006).

We note that not all reports of Bt cotton performance in India are positive. A survey of growers in the regions of Maharashtra and Andhra Pradesh found that Bt hybrids yielded 15–17% less than two conventional (non-counterpart) hybrids, causing economic losses for farmers (Sahai and Rahman 2003). The authors also claimed that Bt hybrid cotton failed to provide protection against bollworms and produced fewer bolls and lower quality lint, yet their paper did not contain any data supporting these claims. Other studies have concluded that Bt cotton failed in India (see Shiva and Jafri 2003, Qayum and Sakkhari 2004, Qayum and Sakkhari 2005). However, because these results were not published in a peer-reviewed source, they are not considered here. Nevertheless, the idea of Bt cotton failure in India has sparked additional studies examining Bt hybrid performance in greater detail.

The simplest explanation for the reported failures of Bt hybrids in some regions in India may be that researchers compared hybrids with very different genetic backgrounds, which may have confounded the results. As noted above (see *China*), it is most useful to compare transgenic and conventional cotton plants that have the same genetic background. Some Bt hybrids may be poorly suited to certain unfavorable growing conditions compared to locally adapted hybrids (see *Development of commercial cultivars*; Qaim et al. 2006). Sahai and Rahman (2003) compared two Bt hybrids to two unrelated non-Bt hybrids. One of these non-Bt hybrids (Banni or Bunny) has outperformed many other popular conventional hybrids (Qaim et al. 2006). Furthermore, Sahai and Rahman (2003) admit that the non-Bt counterparts to the Bt hybrids used in their study do not perform well in these regions. Thus, the genetic background of the Bt hybrids used in this study probably caused the failure rather than the Bt genotype itself. This illustrates the need to choose appropriate genetic backgrounds when developing hybrids. The continued development of new Bt hybrids that are adapted to local growing conditions is necessary to provide growers with high-yielding Bt hybrids. As mentioned previously, many more Bt hybrids (including a Bt Banni hybrid) have been developed recently. These new hybrids could perform better in regions like Andhra Pradesh. We are not aware of published research on the performance of these new Bt hybrids.

Low performance of Bt hybrids in regions such as Andhra Pradesh and Maharashtra could also result from regional differences in environmental conditions or insecticide use (Qaim 2003, Qaim et al. 2006). As mentioned previously, soil fertility and water availability are common problems for cotton growers in India (Jayaraman 2002, Qaim 2003, Huesing and English 2004). Further, farmers in these regions typically experience lower yields for both Bt and non-Bt cotton than farmers in more environmentally favorable regions (Qaim 2003, Bennett et al. 2006, Qaim et al. 2006). In addition, farmers in Andhra Pradesh typically use more insecticides than farmers in other areas and may not lose as much of their yield to insect damage (Qaim et al. 2006). Hence, for reasons described previously (see *China*), Bt hybrids may not significantly increase yields in Andhra Pradesh (Qaim et al. 2006).

In addition, levels of bollworm infestation vary annually and can affect the performance of Bt cotton (Qaim et al. 2006, Sharma and Pampapathy 2006). Higher bollworm infestations usually occur with increased rainfall, and years with higher bollworm infestations typically result in greater yield gains of Bt hybrids versus conventional cultivars (Qaim 2003, Qaim et al. 2006, Sharma and Pampapathy 2006). Moreover, because rain renders insecticides less effective by washing the chemicals off plants, Bt hybrids have an extra advantage over conventional cotton by having an innate resistance to bollworms. On the other hand, in years of low bollworm infestations, the number of larvae on unsprayed Bt hybrid plants can be similar to numbers on conventional cotton, although Bt hybrids receive less bollworm damage to squares and bolls (Sharma and Pampapathy 2006). Thus, the difference in yield between Bt and non-Bt hybrids may be reduced in years of low infestations (Sharma and Pampapathy 2006).

Another potential reason for the low performance of hybrid Bt cotton may be the use of uncertified or saved seed, which can be less effective against target pests. The extent to which farmers save Bt seed is not precisely known. However, between 1998 and 1999, 33% of seed used to grow fiber crops (including cotton) was saved from previous years (Gadwal 2003). Some seed companies in India have created unofficial Bt hybrids that are not approved by the Indian government (Bennett et al. 2005). Because these hybrids have not been stringently field tested, they may not perform as well as official hybrids. Moreover, if seeds from official (or unofficial) hybrids are saved after harvests, they are unlikely to perform as well as the hybrids themselves. Because hybrid cotton plants only contain one copy of the transgene, approximately 25% of seeds from self-fertilized or interbred hybrid individuals will not carry the *cry1Ac* transgene. Thus, saved seed from hybrid plants will not provide the same level of protection against insects as the previous year's plants. Although saved seeds and unofficial Bt hybrids yielded less than official hybrids, fields planted from saved seeds and unofficial Bt hybrids still out-yielded non-Bt plants, which illustrates the high efficacy of Cry1Ac against cotton pests (Bennett et al. 2005).

### South Africa

Cotton agriculture in South Africa is split between large commercial farms and resource-poor smallholders (Huesing and English 2004, Gouse et al. 2004, Raney 2006). The majority of smallholder growers are located in the Makhathini Flats in the KwaZulu Natal province (Thirtle et al. 2003). This region is the least developed in South Africa, and the average landholdings are less than five hectares (Thirtle et al. 2003, Huesing and English 2004. Bennett et al. 2004, Hillocks 2005, Morse et al. 2005. Morse et al. 2006, Hofs et al. 2006b). The region, like much of South Africa, is largely rain-fed, although irrigation is available in some areas (Thirtle et al. 2003, Morse et al. 2005, Morse et al. 2006, Hofs et al. 2006b). Soil fertility and climatic conditions in South Africa are generally favorable for cotton production (Hofs et al. 2006b), although pest pressure is relatively high (Kirsten and Gouse 2003, Qaim and Zilberman 2003, Morse et al. 2005, Morse et al. 2006). Smallholder growers have access to insecticides, but the application of these chemicals can be labor-intensive (Kirsten and Gouse 2003, Huesing and English 2004, Hofs et al. 2006b). Until the 2002–2003 growing season, smallholders in the Makhathini Flats had access to credit and extension services through a local seed provider (Thirtle et al. 2003, Bennett et al. 2004, Gouse et al. 2005). In 2002, the seed provider, for economic reasons, was unable to continue offering credit to cotton growers (Gouse et al. 2005). As a result, cotton production declined in the region (Hofs et al. 2006b).

Bollworms (*H. armigera, Diparopsis castanea*, and *Earias* spp.) are the most economically important group of cotton pests in South Africa (Green et al. 2003). To improve control of these pests, a transgenic cotton cultivar containing *cry1Ac* (Bollgard, Monsanto) was commercially released in South Africa in 1998 (Morse et al. 2006). Currently, South African farmers have access to several Bollgard and Bollgard II cotton varieties, and Bt hybrids are rarely, if ever, planted. In the 2003–2004 growing season, transgenic insect resistant cotton comprised 81% of the national production, with some areas reaching 90% adoption (Thirtle et al. 2003, Morse et al. 2005, Hofs et al. 2006b).

Most studies examining the performance of Bt cotton in South Africa have been conducted in the Makhathini Flats (Thirtle et al. 2003, Bennett et al. 2004, Gouse et al. 2005, Morse et al. 2005, Morse et al. 2006, Hofs et al. 2006b). Studies of cotton performance in this region over multiple seasons typically demonstrated increased yields of 22–85% for Bt cultivars over isogenic and non-isogenic conventional cultivars (Kirsten and Gouse 2003, Thirtle et al. 2003, Bennett et al. 2004, Morse et al. 2005, Morse et al. 2006). The largest yield gains for Bt cultivars were observed during seasons of poor growing conditions (Kirsten and Gouse 2003, Gouse et al. 2005, Morse et al. 2005). In addition, due to less staining caused by bollworms, the lint of transgenic cotton was reported to be of higher quality than the lint of non-isogenic non-transgenic cultivars (Morse et al. 2006). Several studies in South Africa and India document that transgenic cotton had higher early boll retention than non-transgenic cotton, most likely as a result of greater protection against insect damage in transgenic varieties (Hofs et al. 2006a, Hofs et al. 2006b, Hebbar et al. 2007a, Hebbar et al. 2007b). Higher early boll retention can lead to early senescence and early harvesting, particularly when water is limiting as in rain-fed areas (Hebbar et al. 2007a, Hebbar et al. 2007b).

However, two studies of large commercial farms and smallholder farmers in the Makhathini Flats reported that although yields tended to be higher for Bt cotton cultivars than isogenic conventional cotton, these trends were not always statistically significant (Hofs et al. 2006a, Hofs et al. 2006b). The authors suggested that earlier studies showing a significantly greater yield of Bt cotton relied on data collected from the local credit and seed provider that was not a truly random sample of farmers in the region. This does not discredit the valuable gains from adopting Bt cotton that farmers may receive when they have access to credit (necessary to purchase Bt cottonseed) and technical support. However, the situation with the credit provider in the Makhathini Flats illustrates the importance of credit and extension services to Bt cotton adoption and cotton production in general (Huesing and English 2004, Bennett et al. 2004, Gouse et al. 2005, Hofs et al. 2006b). Studies examining the performance of transgenic cotton across a wider range of growing practices could be useful to more comprehensively assess the performance of transgenic cotton in sub-Saharan Africa.

Hofs et al. (2006b) suggested that the most important benefit of growing Bt cotton in South Africa is a reduction in insecticide use. Indeed, Bt cotton adopters used 2–75 times less bollworm-targeting active ingredient than non-adopters from 1998 to 2001 (Morse et al. 2006). This large range in insecticide reduction probably reflects variation in knowledge about Bt cotton. For example, Bt adopters in the first season of adoption (1998–1999) reduced their insecticide use by 50%, while farmers in the following season used almost no insecticides for bollworms (Morse et al. 2006). By the 2000–2001 growing season, insecticide use on Bt cotton had reached a more moderate level of 4.5 times less insecticide active ingredient than isogenic and non-isogenic conventional cotton (Morse et al. 2006). In addition, Morse et al. (2006) found that smallholder farmers growing Bt cotton decreased their use of non-bollworm insecticides by 26–60%. Because Bollgard does not affect non-lepidopteran pests, this trend may be a result of poor understanding about the nature of Bt cotton, or it could indicate a greater abundance of natural enemies in transgenic cotton fields. In addition, Kirsten and Gouse (2003) noted an increase in jassids on Bt cotton plants, possibly as a result of reduced insecticidal sprays for bollworms that also controlled jassids. The economic importance of damage to Bt cotton caused by jassids in South Africa is unknown, although jassids are generally considered easier to control than bollworms (Kirsten and Gouse 2003).

### Factors affecting transgene expression

Since the introduction of commercial transgenic cotton, several researchers have noted seasonal and spatial variation in transgenic toxin content of these plants. Two types of factors are responsible for this variation: plant characteristics and environmental conditions. Most of the research on these factors has been conducted on Bollgard cotton, and unless stated otherwise, the following sub-sections describe observations of Bollgard cotton.

### Characteristics of transgenic cultivars

Three patterns in transgene expression can be attributed to cotton plant characteristics: differences in toxin levels and efficacy between cultivars, between plant parts, and over the growing season. Some Bt cultivars and hybrids contain as much as seven times more Cry1Ac than others, although most varieties differ by less than two-fold (Sachs et al. 1998, Adamczyk and Sumerford 2001, Adamczyk et al. 2001a, Adamczyk and Meredith 2004, Chen et al. 2005a, Chen et al. 2005b, Kranthi et al. 2005, Olsen et al. 2005). In addition, some Bt cultivars lose their toxicity over the growing season 1.2–14 times faster than others (see below; Sachs et al. 1998, Adamczyk and Sumerford 2001, Adamczyk et al. 2001a, Chen et al. 2005a, Chen et al. 2005b, Kranthi et al. 2005). Although it is not yet clear whether the variation in toxicity affects yield (Bryant et al. 2003, Dong et al. 2006), Bt toxin concentration is positively associated with efficacy against bollworms (Adamczyk et al. 2001a, Olsen et al. 2005). Therefore, Bt varieties and hybrids with higher toxin concentrations probably produce higher yields and greater reductions in insecticide use than cultivars with lower toxin concentrations in areas where bollworms are key pests of cotton.

The genetic background of a Bt cultivar affects the amount of toxin produced (Sachs et al. 1998, Adamczyk and Sumerford 2001, Adamczyk and Meredith 2004). However, the mechanisms underlying the interaction between a cultivar's genetic background, transgene expression, and toxin concentration remain unclear. Plants with different genetic backgrounds may vary in levels of transcription factors that are involved in *cry1Ac* expression. For example, if a cultivar produces more *cry1Ac* transcription factors, that cultivar may have higher *cry1Ac* expression than a variety with a different genetic background. Furthermore, the plant hormone auxin increases the activity of CaMV 35S (the promoter of *cry1Ac*; Liu and Lam 1994), and Bt cultivars may vary in auxin production and thus *cry1Ac* expression. It is also possible that cultivars differ in levels of molecules that stabilize *cry1Ac* mRNA or Cry1Ac protein production. Finally, Cry1Ac levels in cotton may be affected by nitrogen metabolism, overall protein production, and interactions with other toxins (see below), which may vary among cultivars because of their genetic background.

The efficacy of Bt cultivars and hybrids can decline over the growing season because of decreasing levels of Cry1Ac (Adamczyk et al. 2001a, Adamczyk et al. 2001b, Bird and Akhurst 2005, Kranthi et al. 2005, Olsen et al. 2005). Cry1Ac levels usually begin dropping as the cotton plants start producing flowers and bolls, and Bt concentrations during the reproductive stage are as low as one-fifteenth the concentration before reproduction (Sachs et al. 1998, Greenplate et al. 1998, Greenplate 1999. Adamczyk and Sumerford 2001, Adamczyk et al. 2001a, Bird and Akhurst 2005, Chen et al. 2005a, Chen et al. 2005b, Olsen et al. 2005). However, the seasonal decline in toxin concentrations varies widely among cultivars (Sachs et al. 1998, Adamczyk and Sumerford 2001, Adamczyk et al. 2001a, Adamczyk et al. 2001b, Kranthi et al. 2005). Some cultivars lose as little as 5% of their toxicity, while others lose as much as 99% during the growing season (Sachs et al. 1998, Chen et al. 2005a, Chen et al. 2005b, Wan et al. 2005). Thus, Bt cultivars showing large reductions in Cry1Ac concentration over the growing season become more susceptible to insect damage, particularly from insects with moderate susceptibility to Cry1Ac such as *Helicoverpa* spp. (Olsen and Daly 2000. Adamczyk et al. 2001a, Kranthi et al. 2005, Olsen et al. 2005). Toxin levels in Bollgard II and VipCot do not change in the same way as Cry1Ac levels in Bollgard (Adamczyk et al. 2001c, Llewellyn et al. 2007a). Cry2Ab concentrations in Bollgard II cotton tend to spike in mid-season before declining (Adamczyk et al. 2001c), while levels of Vip3A remain relatively stable throughout the season (Llewellyn et al. 2007a). Despite the more consistent Vip3A concentrations, Cot 102 plants lose some of their efficacy against *H. armigera* during mid-season (Llewellyn et al. 2007a).

The seasonal reduction in Cry1Ac concentration in Bt cotton could be caused by mRNA instability, declining promoter activity, reduced nitrogen metabolism, lower overall protein production, and toxin interactions. Olsen et al. (2005) suggested that the seasonal decline may be a result of unstable *cry1Ac* mRNA and a reduction in CaMV 35S promoter activity, possibly because of changing auxin levels (see above). However, the expression of *cry1A* with a non-CaMV 35S promoter in the Chinese cultivar GK19 also declined over the growing season, and Cry1A toxin levels were generally lower during the reproductive stages than Cry1Ac levels in Bollgard cotton (Wan et al. 2005). This suggests that factors besides the promoter affect toxin levels. Reductions in Bt toxin production as plants age may result from a decline in overall protein production (Sachs et al. 1998, Chen et al. 2005a, Olsen et al. 2005). Reduced protein production is likely an effect of changes in nitrogen metabolism that occur as the plant shifts more nutrient resources to its reproductive tissues (Chen et al. 2005a). Moreover, when commercially-produced Cry1Ac was mixed with pre-square and fruiting non-Bt cotton leaves and fed to *H. armigera* neonates, neonate survival was 14–726 times higher on the fruiting leaf mixture than the pre-square mixture (Olsen and Daly 2000). Thus, some phytochemicals produced by cotton plants may directly interfere with the efficacy of Bt toxins. Concentrations of condensed tannins (i.e., anti-herbivory protein-binding molecules) increased in cotton as the plant developed (Zummo et al. 1984). These tannins can interfere with Cry1Ac efficacy by deterring insect feeding or binding to Cry1Ac itself (Navon et al. 1993, Olsen et al. 1998).

Finally, Cry1Ac concentration varies among plant tissues in transgenic cultivars and hybrids (Greenplate et al. 1998, Greenplate 1999, Adamczyk et al. 2001a, Gore et al. 2001, Kranthi et al. 2005, Wan et al. 2005). While seeds have high concentrations of Cry1Ac (Adamczyk and Sumerford 2001), leaves typically contain 1.8–19 times more Cry1A or Cry1Ac than reproductive parts such as squares, flowers, and boll maternal tissue (Greenplate et al. 1998, Greenplate 1999, Adamczyk et al. 2001a, Kranthi et al. 2005). Toxin levels in newlyformed bolls were particularly low and sank below the concentration necessary for adequate protection against bollworms during the growing season (Adamczyk et al. 2001a, Kranthi et al. 2005). It remains unclear why different tissues produce different levels of Bt proteins, although the possible explanations are similar to those discussed previously. Because plant tissues have different functions that require specific proteins, the type and level of gene expression varies widely between tissues. Although every cell contains all the genes a plant needs, only the genes needed for the function of leaf tissue, for example, will be expressed in the leaves. Thus, plant tissues may vary in factors that affect transgene expression such as promoter activity, mRNA stability, nitrogen metabolism, overall protein production, and condensed tannin concentrations simply because these factors are needed in some tissues more than others.

Although many of the details concerning Cry1Ac expression and toxin content remain unknown, it is clear that the genetic background of a transgenic plant plays a significant role in Bt toxin production and efficacy against insect pests. For this reason, careful plant breeding and testing are necessary to optimize the efficacy of transgenic cotton. Not only should breeders rigorously select the genetic background of their transgenic cotton plants, but these plants should undergo stringent laboratory and field testing to ensure optimal transgene expression and efficacy under local growing conditions.

### Environmental factors

Several environmental factors are known to alter Bt concentrations in cotton (reviewed by Dong and Li 2007), but many other aspects of the environment have yet to be examined. Here we describe two of the more well-studied environmental factors that affect Bt concentrations in cotton: nitrogen and temperature.

Nitrogen is an important component of amino acids, which are the building blocks of proteins. This suggests that nitrogen availability could affect Bt protein production. Although this hypothesis has not been fully evaluated, Bt cotton (Bollgard with Cry1A) had significantly higher (19–36%) leaf nitrogen contents than conventional isogenic cultivars, suggesting a higher uptake of nitrogen in Bt than conventional cotton (Coviella et al. 2002, Chen et al. 2005a). Furthermore, increasing nitrogen fertilizer raised concentrations of Bt toxins (Coviella et al. 2002), and the seasonal decline in Cry1Ac concentration was somewhat mitigated by nitrogen fertilizer (Pettigrew and Adamczyk 2006). Cry1A cotton plants may also have more active nitrogen metabolisms than isogenic conventional cultivars (Chen et al. 2005a).

It remains unclear how the insertion of *cry1A* or *cry1Ac* genes into the cotton genome may cause higher nitrogen contents and metabolisms. Chen et al. (2005a) suggested that the transgenes may indirectly cause higher levels of vegetative growth at the expense of reproductive output because the plant's natural balance between nitrogen and carbohydrate metabolisms is changed as a result of Bt toxin production. Results from performance studies conducted in China, India and South Africa (see *Performance of transgenic cotton in developing countries*) indicate that differences in nitrogen requirements between Bt and non-Bt cotton had little effect on yield, compared to the yield gains resulting from increased protection against pests in Bt cotton. However, more research is needed to fully explore the consequences of these potential differences in nitrogen requirements between Bt and non-Bt cotton. Will the relative performance of Bt cotton be maintained across the range of soil nitrogen contents typically encountered in developing countries? Will the applications of nitrogen fertilizer that result in higher toxin concentrations and more vegetative growth increase or decrease yield in developing countries?

Temperature can also alter the concentration and efficacy of Cry1A and Cry1Ac in transgenic cotton. Leaves collected from pre-square Bollgard plants grown at 22–32°C were significantly more toxic to *H. armigera* than leaves grown at 14–24°C (Olsen et al. 2005). Similar changes in toxicity also occurred in plants exposed to high or low temperatures for only seven days (Olsen et al. 2005). The Cry1Ac concentration in leaves did not differ between plants exposed to high and low temperatures, indicating another trait affected efficacy. However, Chen et al. (2005b) observed a significant decline in Cry1A concentration in plants exposed to short bursts of high temperatures (37°C) compared to plants maintained at constant temperatures (25–32°C). The reduction in Cry1A also paralleled a decline in amino acid synthesis and an increase in protein degradation, suggesting that high temperatures disrupt nitrogen metabolism in transgenic cotton plants. These two studies are not necessarily contradictory. Changes in temperature may have primarily affected the production of the plant's natural defenses at moderate temperatures (below 32°C), while higher temperatures (37°C) may have stressed the plants and changed their nitrogen metabolism. It remains unclear whether high temperatures could reduce the efficacy of Bt toxins to the extent that yields are significantly affected. Studies conducted in Arizona where temperatures are often above 40°C suggest that high temperatures did not impair performance of Bt cotton (Tabashnik et al. 2000, Cattaneo et al. 2006).

### Gene flow between transgenic and conventional cotton

Transgenic cotton offers many important economic and environmental benefits (see *Performance of transgenic cotton in developing countries*). Nevertheless, the potential for transgenic crops to genetically contaminate related plants has generated international concern (Smyth et al. 2002, Marvier and Van Acker 2005). Such contamination, known as “gene flow,” may occur via cross-pollination between transgenic and non-transgenic plants (i.e., wild cotton relatives or conventional cotton varieties), emergence of volunteer transgenic plants in non-transgenic fields, or inadvertent mixing of seed during planting, processing, or marketing (Smyth et al. 2002). Gene flow between cultivars is of particular concern for the seed production industry, because transgenes may accumulate in the conventional seed supply over time. The result is an inability of growers or retailers to purchase a transgene-free product and an inability of biotechnology firms to regulate use of their product. While most controversy has centered on the contamination of non-transgenic cultivars by transgenic crops, it is also in the best interest of seed producers to limit contamination of transgenic varieties by their conventional counterparts, which could result in genetic dilution of the desired trait.

### Consequences of gene flow

In the United States and Canada, the contamination of conventional crop varieties by their transgenic counterparts has resulted in product recalls (Lin et al. 2003, Mellon and Rissler 2004), lawsuits (Vermij 2006), and, in an extreme case, the collapse of the market for organic canola production in western Canada (Smyth et al. 2002). In the United States, organic agricultural products are not permitted to contain transgenes (Mellon and Rissler 2004). Thus, segregation of transgenic and non-transgenic cultivars is a concern for organic cotton farmers in developing countries (Hillocks 2005, Matthews and Tunstall 2006).

The European Union has a low tolerance for transgene presence in conventional agricultural commodities. In response to a contamination event in Canada involving transgenes in conventional canola, which resulted in the exportation of contaminated seed to Europe, France destroyed 600 hectares of canola grown from the imported seed, and Sweden prohibited marketing of the canola in Europe (Smyth et al. 2002). Therefore, the continued segregation of transgenic and non-transgenic cultivars is vital for growers intending to export to Europe.

The contamination of fields of transgenic cotton by conventional cotton plants could reduce insecticidal properties of transgenic cultivars (see *Performance of transgenic cotton in developing countries*). Furthermore, the contamination of non-transgenic refuges by transgenic plants, or the contamination of fields of transgenic cotton by conventional cotton, could reduce the effectiveness of the refuge strategy for delaying insect resistance (see *Management of pest resistance to transgenic cotton*). The sections below address cross-pollination of conventional cotton by transgenic cotton, limiting unwanted gene flow from transgenic cotton, and outcrossing of wild cotton with transgenic cotton.

### Cross-pollination of conventional cotton by transgenic cotton

Compared to other crops, relatively little attention has focused on gene flow between cotton varieties. The scarcity of cotton outcrossing research may be attributable to the reputation of cotton as a primarily self-pollinating crop. Although cotton primarily self-pollinates, it is readily outcrossed by insects, particularly bees (Free 1970). Outcrossing of non-transgenic cotton plants by transgenic cotton plants occurs when pollen is carried from a flower on a transgenic plant to a flower on a non-transgenic plant, resulting in fertilization of one or more ovules in the recipient flower. When outcrossing involves a conventional cultivar and a true-breeding transgenic cultivar (see *Development of transgenic cotton cultivars*), out-crossing produces hemizygous seeds and offspring with a single copy of the transgene. Because the production of Bt toxins is dominantly inherited, hemizygous seeds produce high levels of Bt toxin (Sachs et al. 1998, Zhang et al. 2000b, Heuberger et al. 2008a). While conventional cultivars may become contaminated via outcrossing, emergence of volunteer plants, or accidental mixing of seed, outcrossing has received the most attention. Investigating the other mechanisms of transgene entry would require large-scale study of commercial cotton fields and, to our knowledge, no such study has taken place.

Studies conducted in the United States, China, and Australia, have examined gene flow from Bt cotton to non-Bt cotton over various distances. Most have found low levels of outcrossing, and sharp declines in outcrossing as distance into adjacent plots of non-Bt cotton increased. In Mississippi, up to 5.7% of seeds were outcrossed in non-Bt cotton rows adjacent to a 136m × 30m test plot of Bt cotton, and outcrossing decreased to less than 1% of seeds at 7m from the Bt plants (Umbeck et al. 1991). In China, rows of non-Bt plants adjacent to a 6m2 plot of Bt cotton were outcrossed at rates up to 8.2% of seeds, but outcrossing at 50m from the Bt plants was undetectable (Zhang et al. 2005b). In experimental plots in Australia, Llewellyn and Fitt (1996) reported up to 0.9% outcrossed seeds in rows of non-Bt cotton adjacent to a block of approximately 3,000 Bt cotton plants, and rates declined to less than 0.03% at 16m from the Bt plants (Llewellyn and Fitt 1996). In a more recent large-scale study, Llewellyn et al. (2007b) found outcrossing rates of up to 30% of seeds in non-Bt cotton rows adjacent to Bt cotton.

### Limiting unwanted gene flow from transgenic cotton

Because outcrossing declines dramatically with distance, buffers can be used to reduce unwanted gene flow into conventional cotton fields grown for seeds (California Crop Improvement Association 2007). A buffer zone is the area bordering a field on all sides and defined by a fixed distance from the edge. Seeds from plants occurring in the buffer zone, which are expected to receive the majority of gene flow from surrounding fields, are not harvested at the end of the season. In California, growers of cotton seed receiving the “registered” designation for seed purity must separate their cotton fields from other cotton varieties by a minimum of 200m and must set aside 6m of unharvested buffers around the fields (Hutmacher and Vargas 2006, California Crop Improvement Association 2007).

Buffers of conventional cotton can also be planted around transgenic fields to limit gene flow from the transgenic fields. This method has been widely used for limiting gene flow from experimental transgenic varieties (Llewellyn and Fitt 1996, Zhang et al. 2005b, United States Environmental Protection Agency 2006). Based on their findings in experimental plots in Australia, Llewellyn and Fitt (1996) suggested that 20m buffer zones are generally adequate. In China where higher outcrossing levels were measured, Zhang et al. (2005b) recommended buffers of 60m.

Because cotton is outcrossed by pollinating insects, fields with abundant pollinators can have increased outcrossing. This was demonstrated experimentally in a study conducted in California with transgenic herbicideresistant cotton and non-transgenic cotton (Van Deynze et al. 2005). At a site where pollinators were not introduced, non-transgenic cotton plants occurring within 0.3m of herbicide-resistant plants were less than 2% outcrossed. In contrast, at a site where four commercial honeybee hives were introduced, non-transgenic cotton plants within 0.3m of herbicide-resistant plants were up to 7.6% outcrossed (Van Deynze et al. 2005). Similarly, Llewellyn et al. (2007b) observed more outcrossing in cotton fields of northern Australia where bee abundance was relatively high compared to fields in eastern Australia where bees were scarce. Because cotton fields containing abundant pollinators are subject to greater outcrossing than typical fields, the width of buffers should be increased for these fields to achieve the desired level of gene containment (Llewellyn et al. 2007b).

While buffers are a popular and well-tested method for limiting gene flow, certain factors may limit their success. In an unusual scenario where non-Bt fields were contaminated by adventitious Bt plants, Heuberger et al. (2008b) did not observe the expected decline in outcrossing with distance into non-Bt fields. The study, conducted in Arizona, showed that some purchased bags of non-Bt cotton seed were contaminated at low rates by adventitious Bt cotton seeds. In experimental plots where the planted seed was contaminated at a rate of 8%, rows that were 20m from the field edge had no less Bt outcrossing than rows at the field edge. Adventitious Bt cotton plants in the non-Bt plots could have outcrossed surrounding non-Bt plants and produced the unexpectedly high outcrossing rates near the center of the non-Bt plots (Heuberger et al. 2008b). Therefore, growers who use buffers should make certain that the buffer zone is free of contamination.

In addition to buffers, the utility of crop-free or “barren” zones between fields has been examined for reducing cross-pollination between transgenic and non-transgenic crops (Llewellyn et al. 2007b). The problem with this strategy is that pollinators readily move across bare ground between fields over large distances (Llewellyn et al. 2007b). Bumblebees and honeybees, which are known pollinators of cotton (Free 1970), have been observed foraging at distances up to 200m or several kilometers from their nests, respectively (Osborne et al. 1999). Thus, barren zones may be less efficient than cotton buffers for reducing the movement of pollen carried into a field by insects. Llewellyn et al. (2007b) reported that barren zones of 100m or less were ineffective at limiting gene flow between transgenic and non-transgenic cotton fields. Similarly, in a survey of 12 non-Bt cotton fields that were 10–55m from Bt cotton fields, no significant association occurred between outcrossing rate and the distance between fields (Heuberger et al. 2008b).

### Outcrossing of wild cotton with transgenic cotton

Outcrossing of wild plant species with their transgenic relatives could endanger the genetic integrity of native species and introduce traits that promote weediness (Ellstrand 2001, Munster and Wieczorek 2007). Therefore, growers of transgenic cotton should exercise caution in regions with native cotton species that are genetically compatible with transgenic cultivars. For example, the United States prohibits the commercial sale of Bt cotton in Hawaii and in some parts of Florida, because wild cotton species that are genetically compatible with Bt cotton occur in those regions. As an exception, Bt cotton test varieties and nursery stock can be produced in Hawaii, but must be surrounded by 12–24 rows of non-Bt cotton, depending on field size, and cannot be planted within 0.25 mile of *Gossypium tomentosum*, the genetically compatible native species (United States Environmental Protection Agency 2006).

### Implications for use of transgenic cotton in developing countries

According to the Action Group on Erosion, Technology and Concentration (ETC, http://www.etcgroup.org/en/), over a billion farmers plant their fields with farm-saved seed (Shand 2002). Many are subsistence farmers in developing countries who cannot afford to purchase seed. Because the seed is continually saved and replanted on these farms, the effects of recurrent gene flow could accumulate over multiple seasons. Gaines et al. (2007) investigated contamination of farm-saved wheat seed by non-transgenic imidazolinone-resistant (IR) wheat in Colorado. They compared non-IR seed from certified seed lots with farm-saved seed, and found that farm-saved seed contained significantly more IR contamination (0–11.3%) than certified seed (0–4.2%). Because farmsaved seed is particularly vulnerable to contamination, precautions to safeguard local conventional cotton varieties should be taken in developing countries where transgenic cotton varieties are introduced. Conserving seed of local cotton varieties in seedbanks, and providing affordable, certified seed of local varieties to subsistence farmers, could prevent the loss of these genetic resources. Similarly, providing affordable, certified seed of transgenic cotton varieties could help preserve the insecticidal properties of transgenic cotton and aid in the sustainable management of pest resistance. Measures should also be implemented to preserve the integrity of organic cotton.

### Non-target effects of transgenic crops

*Bacillus thuringiensis* is a ubiquitous spore-forming bacterium naturally present in soil, dead insects, stored products, bird and bat nests, pond water, on the surface of numerous cultivated and uncultivated plants, and in other environments (Bernhard et al. 1997). Bt sprays have been used for decades in conventional and organic agriculture, in forestry, and for the control of disease vectors (Walker et al. 2003). While Bt sprays contain a mixture of cultured spores and vegetative stages, a few insecticidal crystal (Cry) and one vegetative (Vip) proteins are the basis of defense against insects in nearly all commercially grown transgenic cotton (Table 1). Past experience with Bt sprays indicates that such proteins kill target pests without causing significant harm to people, wildlife, and most non-target arthropods (Federici 2003).

Most Bt toxins produced by transgenic cotton are similar to toxins found in Bt sprays (Federici 2003, Mendelsohn et al. 2003). However, the concentration of Bt toxins in cotton remains high for months, whereas Bt sprays degrade in days. Moreover, the incorporation of plant residues with Bt proteins into the soil represents a novel route of exposure for soil organisms. These differences have caused concerns about the potential negative impacts of transgenic cotton on non-target organisms.

Risk assessment of non-target effects has primarily focused on impacts of transgenic cotton on organisms providing ecological services, such as parasitoids and predators, pollinators, and soil organisms involved in decomposition and nutrient cycling (O'Callaghan et al. 2005, Romeis et al. 2006). The traditional tiered approach used to assess the risks of synthetic insecticides was adapted for assessment of transgenic crops (Poppy 2000, Button et al. 2003, Hill 2005). Tier one considers acute toxicity of toxins from transgenic crops or plant material in the laboratory. Tier two involves experiments conducted under “semi-field” conditions (e.g., herbivores and natural enemies caged with plants, earthworms kept in containers with different soil types). Tier three examines ecological interactions affecting non-target organisms in the field. The first two tiers are useful to establish causal relationships between the presence of toxins and non-target effects (Romeis et al. 2006). The third tier is required to evaluate impacts in agroecosystems. For example, laboratory studies revealed that ingestion of pollen from Bt corn killed monarch butterfly larvae. However, field studies revealed that exposure of monarch populations to Bt pollen from most commercial cultivars was too low to have significant negative impacts (Sears et al. 2001).

Cotton is typically protected from pests with synthetic insecticides (Fitt 1989, Luttrell et al. 1994). Many of the older, broad-spectrum insecticides with negative impacts on non-target arthropods are still used in cotton. Because transgenic cotton controls certain key pests and has the potential to significantly reduce insecticide use (see *Efficacy of transgenic cotton against arthropod pests*; Carpenter et al. 2002, Shelton et al. 2002, Wu and Guo 2005, Cattaneo et al. 2006), realistic field assessments of the non-target effects of transgenic cotton have to take into account patterns of insecticide use in transgenic and conventional cotton (Cattaneo et al. 2006, Marvier et al. 2007, Sisterson et al. 2007).

Below, we review current findings on the impacts of transgenic cotton on non-target organisms. We primarily focus on studies assessing impacts of Cry1Ac cotton (Table 1), as few studies on other transgenic cultivars (Hagerty et al. 2005, Whitehouse et al. 2007) have been published in English (but see papers in Chinese cited in Wu et al. 2003a and Wu and Guo 2005).

### Natural enemies

Negative impacts on natural enemies could occur through direct ingestion of Bt toxins in hosts, prey, or plant material, or changes in the quality or abundance of Bt-intoxicated hosts or prey. Direct toxic effects of Bt toxins on parasitoids and predators appear rare (Romeis et al. 2006). Rather, negative effects occur when natural enemies feed on Bt-intoxicated hosts or preys that are susceptible to Bt (Romeis et al. 2006). Thus, negative effects of Bt cotton on parasitoids and predators mainly involve a reduction in nutritional quality or abundance of hosts or prey.

Because Bt crops can greatly reduce populations of target lepidopteran pests and negatively affect populations of related species (see *Efficacy of transgenic cotton against arthropod pests*), it is not surprising that decreased abundance or decreased parasitism by natural enemies specializing on lepidopteran pests have occurred in Bt cotton (Wu and Guo 2005, Sisterson and Tabashnik 2005, Romeis et al. 2006). Regional extinction of specialized parasitoids could occur if most of the diet of their hosts was provided by Bt cotton (Sisterson and Tabashnik 2005). Such extinctions could reduce the efficacy of biological control in refuges (see *Management of pest resistance to transgenic cotton*) but would have little effect on the control of key pests in Bt cotton, as long as such pests do not evolve resistance to Bt.

In the absence of insecticide sprays, the abundance of a few generalist predators declined in Bt cotton compared to non-Bt cotton (Naranjo 2005a, Whitehouse et al. 2005). Moreover, a meta-analysis of 42 experimental field studies involving Cry1Ac cotton showed that the abundance of non-target arthropods was slightly but significantly lower in unsprayed Bt cotton compared to unsprayed conventional cotton (Marvier et al. 2007). The reasons for this difference have been hypothesized to result from a reduction in the abundance of lepidopteran insects that are important resources for non-target arthropods. Nevertheless, observed levels of reductions in predator abundance appear to have little functional significance. In a six-year study, the abundance of 5 out of 22 predator taxa was reduced by about 20% in unsprayed Bt cotton compared to unsprayed non-Bt cotton (Naranjo 2005a). However, predation rates were similar in both types of cotton (Naranjo 2005b), suggesting that predation rates can be sustained despite declines in abundance of certain predators (Sisterson et al. 2004a, Naranjo 2005b).

When non-Bt cotton was treated with more insecticides than Bt cotton, the density of predators was significantly greater in Bt than in non-Bt cotton (Naranjo 2005a, Whitehouse et al. 2005, Wu and Guo 2005, Sisterson et al. 2007). However, the abundance of non-target arthropods did not always differ between Bt and non-Bt cotton in such situations (Cattaneo et al. 2006, Marvier et al. 2007). The increased abundance of predators in some cases resulted in higher predation rates in Bt than non-Bt cotton (Head et al. 2005). For example, a reduction in insecticide use in Bt cotton compared to non-Bt cotton was associated with greater abundance of predators and better control of the cotton aphid in the middle and end of the growing season in China (Wu and Guo 2003).

### Pollinators

Ingestion of pollen, nectar, and resin from Bt crops, or ingestion of purified Bt toxins did not have negative effects on honeybees and bumblebees (Malone and PhamDelègue 2001, O'Callaghan et al. 2005). Moreover, bee foraging behavior did not differ significantly between Bt and non-Bt crops. Thus, Bt toxins (at least the Cry proteins) from Bt cotton are considered unlikely to affect bees (Malone and Pham-Delègue 2001, Hanley et al. 2003, Liu et al. 2005, O'Callaghan et al. 2005, Babendreier et al. 2007). Nevertheless, some Bt cotton cultivars used in China produce the cowpea trypsin inhibitor (Table 1). Several studies indicate that this protease inhibitor could have negative impacts on bee health, development, and learning ability (Malone and Pham-Delègue 2001, Liu et al. 2005, Babendreier et al. 2007).

### Soil organisms

Soil organisms are essential for nutrient cycling and the maintenance of soil organic content and stability. These processes could be particularly important in sustaining agricultural productivity in developing countries where farmers may have limited resources to amend soils. Contrary to other transgenic crops (e.g., corn, potato, rice), Bt cotton does not release Bt proteins from root exudates (Saxena et al. 2004). Thus, Bt proteins in the soils of cotton fields originate from plant material incorporated in the soil, mainly after harvest. Bt proteins bind to clay and humic acid and conserve insecticidal activity for long periods (O'Callaghan et al. 2005). Although degradation of Bt toxins in soils is highly variable and not well understood (O'Callaghan et al. 2005), Bt proteins appear less persistent in soils of cotton fields (e.g., maximum of 90 days; Head et al. 2002) than in soils of corn fields (e.g., maximum of 200 days; Zwahlen et al. 2003).

Degradation of Bt plants (rice, tobacco, canola, cotton, corn, and potato) in the soil was generally slower than the degradation of non-Bt plants (Mores et al. 2005). Reasons for slower degradation of Bt plants were unclear but unlikely due to the presence of Bt toxins. Higher lignin contents of Bt plants presumably arising from indirect effects of genetic transformation were implicated, although other differences in plant composition could be involved (Flores et al. 2005, Poerschmann et al. 2005). Slower decomposition rates of Bt than non-Bt cotton could have fitness consequences for some soil organisms (Zwahlen et al. 2003, Clark and Coats 2006).

Most studies of non-target effects on soil organisms have been conducted with crops releasing Bt toxins through root exudates rather than with Bt cotton. Organisms such as earthworms, isopods, collembolans, nematodes, fungi, protozoa and bacteria were assessed. Overall, little or no adverse effects of Bt crops on soil organisms were apparent (Devare et al. 2004, O'Callaghan et al. 2005, Clark and Coats 2006, Bitzer et al. 2006). However, communities of soil organisms and their environment are highly variable and difficult to study, and the ecological relevance of slower decomposition of Bt than non-Bt plants remains unclear. More work would be helpful to obtain a better understanding of the potential impacts of Bt cotton on soil organisms (Mores et al. 2005, O'Callaghan et al. 2005).

### Human and livestock safety

Although this review focuses on the agronomic and ecological implications of transgenic cotton, human and livestock safety has been an issue of concern. Human health assessment of Bt crop products involves studies of *in vitro* digestion, acute toxicity, and allergenicity (Shelton et al. 2002, Mendelsohn et al. 2003). Many Cry proteins (including Cry1Ab, Cry1Ac, Cry3A, and Cry1F) breakdown to undetectable fragments within seven minute of exposure to simulated gastric fluids, indicating that many Bt proteins are unstable in the human stomach (Betz et al. 2000, United States Environmental Protection Agency 2001, Mendelsohn et al. 2003). Acute oral toxicity studies with mice have found no evidence of Cry protein toxicity (Betz et al. 2000, United States Environmental Protection Agency 2001, Shelton et al. 2002, Mendelsohn et al. 2003). No incidents of food allergies to Bt proteins have been reported in spite of their widespread consumption (United States Environmental Protection Agency 2001).

Bt proteins from cottonseed are destroyed during the oil refining process (Shelton et al. 2002). Numerous studies assessing the effects of feeds from Bt and non-Bt crops (primarily maize and potatoes) on beef cattle, sheep, pigs, laying hens, broiler chickens, and growing and laying quails, have found no significant difference in digestibility, feed intake, health, performance, and quality of food of animal origin (Flachowsky et al. 2007).

### Management of pest resistance to transgenic cotton

Resistance to a transgenic cotton cultivar is a genetically based decrease in the frequency of individuals susceptible to the cultivar in a population that has been previously exposed to the cultivar (Tabashnik 1994a). Laboratory studies have shown that populations of several insect pests can evolve resistance to commercially available Bt crops (Tabashnik et al. 2000, Bird and Akhurst 2004, Jackson et al. 2004a, Huang et al. 2007, Mahon et al. 2007). While no target pests evolved resistance to transgenic cotton in the field during the first five years of commercial use (Tabashnik et al. 2003), a review of monitoring data shows that field-evolved resistance to Cry1Ac cotton has been documented in some populations of *Helicoverpa zea* from Arkansas and Mississippi (Tabashnik et al. 2008). Resistance to Cry1Ac in *H. zea* from Arkansas and Mississippi was significantly greater in multiple strains collected from the field in 2002 to 2004 compared to a susceptible laboratory strain (Ali et al. 2006), while no strains collected from the field in 1992 to 1993 (before commercialization of Bt cotton) were resistant (Luttrell et al. 1999). Data from 2005 and 2006 show that resistance has progressed in *H. zea* (Luttrell and Ali 2007).

Monitoring results obtained by measuring field-derived lines of *H. armigera* from each of four regions in India show consistent increases in the LC_50_ of Cry1Ac from 2000–2 to 2004–6 (Gujar et al. 2007). However, Gujar et al. (2007) did not test a laboratory susceptible line simultaneously with the putative resistant lines. Thus, the observed increases in LC_50_, ranging 18 to 119-fold across the regions, could have been caused by decreasing potency of the toxin used in bioassays over time. Similarly, increases in growth rate on Cry1Ac diet were observed from 2002 to 2005 in *H. armigera* lines collected in the Anci and Xijian counties of China (Li et al. 2007). Again, these results are not conclusive because no susceptible line from the laboratory was tested simultaneously with the field-derived lines.

Extensive published monitoring data provide no evidence that resistance to Bt crops has evolved in field populations of *H. virescens, Ostrinia nubilalis* and *Pectinophora gossypiella* in Australia, China, Spain, and the U.S. (Tabashnik et al. 2008). However, the African stem borer, *Busseola fusca*, has evolved resistance to Bt corn producing Cry1Ab in South Africa (van Rensburg 2007), and the fall armyworm, *Spodoptera frugiperda*, has evolved resistance to Bt corn producing Cry1F in Puerto Rico (Reynolds and Matten 2007). Thus, after 12 years of commercial use of Bt cotton, one key pest has evolved resistance to Bt cotton in the field (*H. zea*), and equivocal evidence indicates that a second key pest (*H. armigera*) may be in the process of doing so. We do not explicitly discuss resistance monitoring and remediation methods here. These critical aspects of resistance management have been addressed in several recent publications (Gould et al. 1997, Andow and Alstad 1998, Tabashnik et al. 2000, Venette et al. 2000, Carrière et al. 2001, Andow and Ives 2002, Matten and Reynolds 2003, Fitt et al. 2004, Tabashnik et al. 2003, Tabashnik et al. 2006).

Current transgenic cotton cultivars produce one or two toxins (Table 1). The refuge strategy is commonly used worldwide to delay the evolution of pest resistance to both types of cotton cultivars. Small farmers in certain developing countries could save their seed for producing the next cotton crop (see *Gene flow between transgenic and conventional cotton fields*), resulting in contamination of transgenic cotton seed with non-transgenic seed through time and vice versa. Assuming an absence of significant contamination of cotton seed, we first outline principles that may guide the development of a refuge strategy to delay pest resistance to transgenic cotton, and provide examples of the application of such principles in different agroecosystems. We then consider the implications of contamination of transgenic and non-transgenic cotton seed for resistance management. Finally, we review the effects of transgenic cotton deployment on resistance to synthetic insecticides.

### The refuge strategy

Transgenic cotton cultivars produce concentrations of Bt toxins that are extremely effective against some pests (e.g., *H. virescens* and *P. gossypiella*), but are only moderately effective against others (e.g., *H. armigera* and *H. zea*). For highly susceptible pests, even if there are many alleles that may slightly increase resistance to Bt toxins within insect populations, these alleles may very rarely combine to allow survival on transgenic cotton (McKenzie 1996, Tabashnik and Carrière 2008). On the other hand, individuals that carry one or a few alleles with major effects on resistance could survive on transgenic cotton (McKenzie 1996). Accordingly, it is expected that a single gene with major effects confers resistance to Bt crops producing one toxin, an assumption that was supported in several pests (Tabashnik et al. 1997, Morin et al. 2003, Li et al. 2005, Xu et al. 2005). Furthermore, it is generally assumed for simplicity that such a resistance gene has two alleles (*r* for resistance; *s* for susceptibility). Similarly, it is assumed that two genes each with two alleles (*r1* and *s1; r2* and *s2*) confer resistance to Bt crops producing two toxins that act independently.

The refuge strategy requires that refuges of non-transgenic host plants be planted in or near transgenic cotton fields to promote survival of susceptible pests (United States Environmental Protection Agency 2001, Carrière et al. 2005a, Tabashnik and Carrière 2008). Refuges located outside transgenic cotton fields are called external refuges. Internal refuges may include one or more blocks (i.e., several contiguous rows) of a non-transgenic host planted in a transgenic field, or a single row of a non-transgenic host planted in alternation with rows of transgenic cotton (e.g., one row of non-transgenic cotton for every nine rows of transgenic cotton yields a 10% refuge). Planting transgenic and non-transgenic plants randomly within a field is another way to provide an internal refuge. Decisions about types of refuge used for the management of resistance have been based on mobility of larvae in pests targeted by transgenic cotton (see below, *Management of resistance with contaminated seeds*). Internal refuges have been used to manage resistance in pests with sedentary larvae (e.g., *P. gossypiella*: Carrière et al. 2001, Carrière et al. 2005a), but internal refuges are avoided when pests have mobile larvae that can easily move between plants (e.g., *H. armigera and H. zea*: Gore et al. 2002, Men et al. 2005).

A critical condition for success of the refuge strategy is that abundant susceptible insects from refuges mate with the rare resistant pests surviving on transgenic cotton. If resistance is inherited as a recessive trait, resulting hybrid offspring are killed by transgenic cotton. When the majority of resistant insects that survive on transgenic cotton produce offspring that are killed by that crop, the heritability of resistance is low, which delays the evolution of resistance (Gould 1998, Carrière et al. 2004a, Sisterson et al. 2004b, Tabashnik et al. 2004, Tabashnik and Carrière 2007).

The refuge strategy is based on the general principle that the dominance of resistance depends on the dose of the transgenic toxin (Carrière et al. 2004a). Resistance is often dominant when the dose of a toxin is low, but recessive when the dose is high (Tabashnik et al. 2004). Accordingly, Bt toxin genes incorporated in transgenic cotton were modified to produce high concentrations of Bt toxins (Mendelsohn et al. 2003). Furthermore, genetically-transformed plants producing high concentrations of Bt toxins were used for production of commercial transgenic cultivars (see *Development of transgenic cotton cultivars*). The high concentrations of transgenic toxins are expected to result in recessive resistance in some but not all target pests (Tabashnik et al. 2008).

### Management of pest resistance to cotton producing one toxin

In pests highly susceptible to Bt toxins, toxin concentrations in Bt cotton are high enough to render resistance recessive. Thus, resistance evolution is expected to be substantially delayed even with relatively small refuges (e.g., >5%; Gould 1998, Tabashnik et al. 2008). However, in pests less susceptible to Bt toxins, resistance to Bt cotton is not fully recessive, and large refuges (e.g., > 50%) may be required to efficiently delay the evolution of resistance (Tabashnik et al. 2008).

Non-recessive resistance is a strong indicator for high risk of rapid resistance evolution to cotton producing a single transgenic toxin (Tabashnik et al. 2004, Tabashnik et al. 2008). Resistant lines that can survive on a transgenic crop are necessary to measure whether resistance is recessive. When such lines are not available, the dominance of resistance can be approximated from the efficacy of a transgenic crop against susceptible insects. If a single-toxin transgenic crop kills most (e.g., >99%) of the susceptible insects, resistance is expected to be recessive. However, if a transgenic crop kills <99% of susceptible individuals, resistance is expected to be non-recessive (Tabashnik et al. 2008).

### Management of pest resistance to cotton producing two toxins

Use of plants producing two distinct toxins to delay pest resistance is called the pyramid strategy. The pyramid strategy is expected to be most effective when most susceptible pests are killed by the transgenic crop, resistance to each toxin is recessive, refuges are present, and selection with either of the toxins does not cause cross-resistance to the other (Gould 1998, Roush 1998, Zhao et al. 2005). This strategy is based on the principle that individuals with resistance alleles are killed on two-toxin plants as long as they have a susceptibility allele at either of the resistance loci, a phenomenon called “redundant killing” (Gould 1998, Roush 1998). For example, when resistance to each toxin is recessive, individuals with genotypes *r1r1 s2r2* and *s1r1 r2r2* would survive on singletoxin plants producing toxin 1 and 2, respectively. However, these individuals would be killed on plants producing both toxins.

Fitness costs (see below) keep resistance alleles rare before populations are exposed extensively to transgenic toxins (McKenzie 1996). Thus, the only genotype with high survival on a two-toxin plant (*r1r1 r2r2*) is expected to be extremely rare. Accordingly, when refuges provide many susceptible insects, most offspring from rare resistant individuals will bear at least one *s* allele and will be killed on two-toxin plants, which substantially delays resistance evolution. Because the heterozygous genotypes (*r1s1 s2s2* and *s1s1 r2s2*) carry the majority of resistance alleles after commercialization of a new transgenic crop, a low survival of these genotypes is critical to delay resistance. The pyramid strategy is expected to be efficient when two-toxin plants kill most susceptible insects (e.g., >99.75% mortality of susceptible insects on two-toxin plants, or >95% mortality of susceptible insects on each single-toxin plant: Roush 1998). In this case, even if resistance to a single toxin was not fully recessive (e.g., the mortality of *r1s1* to toxin 1 or *r2s2* to toxin 2 could be 70%), the survival of *r1s1 s2s2* or *s1s1 r2s2* on plants with 2 toxins would be low (i.e., 30% × 5 % = 1.5%), which increases the success of the pyramid strategy (Roush 1998).

Cross-resistance reduces the efficacy of the pyramid strategy because it diminishes redundant killing. Cross-resistance occurs when a genetically-based decrease in susceptibility to one toxin also decreases susceptibility to other toxins. Toxins combined in cultivars are usually chosen because they have low amino acid similarity and bind to different target sites in the larval midgut, two factors expected to minimize the risk of cross-resistance (Ferré and Van Rie 2002). Strong cross-resistance between toxins produced by Bt cotton does not appear common (Tabashnik et al. 2002, Ferré and Van Rie 2002, Xu et al. 2005, Lee et al. 2006, Jackson et al. 2007). However, cross resistance between Cry1Ac and Cry2Aa was documented in some populations of *H. virescens* (Gould et al. 1992, Jurat-Fuentes et al. 2003) and *H. zea* (Burd et al. 2003), and significant cross resistance between other Bt toxins was found in populations of other pests (Moar et al. 1995, Zhao et al. 2001). Thus, whether cross-resistance will significantly affect efficacy of the pyramid strategy in the field remains uncertain.

### Fitness costs and incomplete resistance

Fitness costs occur when fitness in refuges is lower for individuals with resistance alleles than for individuals without resistance alleles. Costs result from indirect effects of resistance alleles; resistance alleles increase fitness on transgenic crops but decrease fitness in the absence of transgenic toxins. Because costs select against resistance in refuges, the evolution of resistance can be delayed substantially, prevented or reversed when costs and refuges are present (Carrière and Tabashnik 2001, Tabashnik et al. 2005, Gould et al. 2006).

The dominance of fitness costs is crucial in the early stages of resistance evolution. If resistance is recessive, individuals heterozygous for resistance alleles are killed by transgenic crops and survive only in refuges. Thus, the fitness of heterozygous individuals relative to that of susceptible individuals in refuges is a key determinant of resistance evolution. Non-recessive fitness costs make fitness in refuges lower for individuals with resistance alleles compared to individuals without. Accordingly, non-recessive fitness costs can strongly favor a decrease in resistance through selection in refuges, even though the rare resistant individuals are favored by selection in transgenic fields (Carrière and Tabashnik 2001, Tabashnik et al. 2005, Gould et al. 2006). With large refuges, recessive costs can also significantly delay or reverse the evolution of resistance (Carrière and Tabashnik 2001, Tabashnik et al. 2005, Gould et al. 2006).

Fitness costs are usually associated with resistance to Bt toxins (Tabashnik 1994a, Ferré and Van Rie 2002, Carrière et al. 2006, Gassmann et al. 2009). Moreover, the magnitude and dominance of costs associated with Bt resistance seems to be frequently affected by environmental conditions, such as variation between host plants (Carrière et al. 2005a, Janmaat and Myers 2005, Raymond et al. 2006, Bird and Akhurst 2007), competition for mates (Higginson et al. 2005), crowding (Raymond et al. 2005), and the presence of natural enemies (Gassmann et al. 2006). This creates an opportunity to manipulate costs to enhance the success of the refuge strategy. For example, field surveys conducted in Australia show that pigeon pea, sorghum and non-Bt cotton are good sources of *H. armigera* for Bt cotton fields (Sequeira and Playford 2001, Bird and Akhurst 2007). In an evaluation of the dominance of costs on these hosts, costs were always recessive on pigeon pea, often additive on cotton, and frequently dominant on sorghum, suggesting that refuges of sorghum and cotton could be better for delaying resistance in *H. armigera* than refuges of pigeon pea. Further study assessing the magnitude and dominance of costs on different host plants or in the presence of natural enemies could improve resistance management through manipulation of refuge quality (Carrière et al. 2005a, Tabashnik and Carrière 2007, Gassmann et al. 2009).

The fitness of resistant individuals is often lower on Bt plants than on their non-Bt counterparts (Carrière et al. 2006), a condition known as incomplete resistance (Carrière and Tabashnik 2001). Unlike fitness costs, incomplete resistance cannot cause reversal of resistance evolution, but it can contribute in delaying resistance (Carrière and Tabashnik 2001, Tabashnik et al. 2005).

### Refuge deployment decisions

Decisions about refuge deployment are based on characteristics of the pest, the transgenic cotton cultivar, the pest-cultivar interaction, and the agroecosystem (Gould 1998, Carrière et al. 2004a, Fitt et al. 2004, Tabashnik et al. 2008). The distance between fields of transgenic cotton and external refuges needs to be less in sedentary pests than in mobile pests. To delay resistance in a pest, every field of transgenic cotton should contain a refuge or have an external refuge at a distance less than the dispersal distance of the pest (Carrière et al. 2004b, Sisterson et al. 2005). Refuges of non-transgenic cotton are required to delay resistance in cotton specialists, while other crops not producing the same transgenic toxin as cotton, or non-cultivated plants, may provide refuges for pests that feed on many hosts. In addition, to increase the occurrence of matings between susceptible and resistant individuals surviving on transgenic crops, concurrent adult emergence in refuges and transgenic cotton fields is critical when non-cotton crops provide refuges (Baker et al. 2008). Strategies used to manage resistance in specific pests are likely to differ geographically, as illustrated below.

### Pink bollworm in Arizona and China

In Arizona, Cry1Ac cotton was the primarily tool for controlling pink bollworm from 1996 to 2006 (Carrière et al. 2001, Carrière et al. 2005b). An eradication program based on extensive use of Bt cotton and the release of sterile moths was initiated in Arizona in 2007. This program drastically changed the refuge requirements for pink bollworm. In what follows, we outline the refuge requirements used in Arizona before 2006 to provide a basis of comparison with requirements currently used in China.

Pink bollworm moths can disperse over long distances, although movement is limited in the presence of suitable cotton plants (Carrière et al. 2001, Carrière et al. 2004b). In Arizona, pink bollworm is an ecological specialist on cotton, and Bt cotton kills virtually 100% of susceptible insects (Tabashnik et al. 2000, Carrière et al. 2003, Carrière et al. 2006). As expected, resistance to Cry1Ac cotton is fully recessive (Carrière et al. 2006). The United States Environmental Protection Agency required a minimum refuge area of 5% of the area of each Bt cotton field planted by farmers for the management of resistance (Carrière et al. 2005b). External refuges at 0.75km or less from Bt cotton consistently act as sources of moths and have the greatest potential to delay the evolution of resistance (Carrière et al. 2004b). Moreover, in-field refuges with a single non-Bt cotton row for every six to ten rows of Bt cotton were commonly used (Carrière et al. 2005b). Bt cotton use reached 84% of cotton acreage in some regions and compliance to the refuge strategy ranged from 70 to 100% of Bt cotton fields across regions (Carrière et al. 2005b). The frequency of pink bollworm resistance to Bt cotton declined from 1997 to 2006 in Arizona (Tabashnik et al. 2005, Tabashnik et al. 2006).

Pink bollworm is also a cotton specialist in the Changjiang River region of China (Wu and Guo 2005). Several Bt cotton cultivars producing Cry1A, Cry1A + CpTI, or Cry1Ac have been used in that region since 1999 (Table 1). However, in contrast to Arizona, density of susceptible pink bollworm larvae on unsprayed Bt cotton is only decreased by 73–89% compared to larval density on unsprayed non-Bt cotton at the end of the growing season (Wan et al. 2004). High survival at the end of the growing season indicates that resistance to Bt cotton in pink bollworm is unlikely to be recessive in the Changjiang River region. This suggests that large refuges of non-Bt cotton would be needed to significantly delay pink bollworm resistance to Bt cotton. However, a refuge strategy has not been explicitly implemented in that region (Wu and Guo 2005). Bt cotton represented 66% of total cotton acreage in China in 2006, suggesting that implementation of a refuge strategy for pink bollworm should be considered (Wu and Guo 2005, Wu 2007).

### Helicoverpa armigera in Australia, China, and West Africa

Because larvae from *H. armigera* can easily move between plants, availability of external refuges has been considered important for managing resistance in this key pest. Bt cotton with a single toxin kills < 99% of *H. armigera* at the end of the growing season (Table 3: Fitt et al. 1998, Wan et al. 2005, Llewellyn et al. 2007a). Data on the efficacy of two-toxin cotton against *H. armigera* are still rare (Table 3), but it is assumed that Bt cotton producing Cry1Ac and Cry2Ab provides good control of *H. armigera* throughout the season (Downes et al. 2007). Experiments conducted in the United States with *H. zea* (a close relative of *H. armigera*) indicate that cotton producing Cry1Ac and Cry2Ab can kill all susceptible larvae (Table 3). However, mortality is not complete in high-infestation years unless cotton is oversprayed with insecticides at the end of the growing season (Jackson et al. 2004b, Hagerty et al. 2005). Until more data are available on field survival of *H. armigera* on two-toxin cotton, a cautious approach for management of resistance would be to use relatively small refuges only when insecticides can be applied to suppress populations in transgenic cotton at the end of the growing season.

Crops other than cotton provide refuges for *H. armigera* in Australia (Fitt 1989, Sequeira and Playford 2001, Baker et al. 2008). Nevertheless, bollworm populations rapidly evolved resistance to synthetic insecticides used in cotton, suggesting that refuges were too rare to efficiently delay the evolution of resistance (Fitt 1989, Fitt and Daly 1990). This prompted a cautious approach for the use of Bt cotton producing the Cry1Ac toxin, which was limited to 30% of the cotton area in Australia from 1996 to 2004 (Downes et al. 2007, Baker et al. 2008). Cotton producing Cry1Ac and Cry2Ab (Bollgard II) was introduced in 2004 and replaced Cry1Ac cotton by 2005. The minimum refuge size for management of *H. armigera* resistance to two-toxin cotton became as low as 5% after 2005 (Downes et al. 2007, Baker et al. 2008). Refuge requirements for Bollgard II depend on the crops used as refuges, based on their capacity to produce susceptible moths: 10 ha of unsprayed conventional cotton/100 ha of Bt cotton; 100 ha of sprayed conventional cotton/100 ha of Bt cotton; 20 ha of unsprayed maize/100 ha of Bt cotton; 5 ha of unsprayed pigeon pea/100 ha of Bt cotton; and 15 ha of unsprayed sorghum/100 ha of Bt cotton (Baker et al. 2008). There is no evidence that *H. armigera* resistance to Cry1Ac and Cry2Ab increased in Australia between 2003 and 2006, although the frequency of recessive alleles conferring high resistance to Cry2Ab seems high compared to the frequency of alleles conferring resistance to Cry1Ac (Downes et al. 2007).

As in Australia, populations of *H. armigera* exploiting cotton in China evolved resistance to synthetic insecticides. Such resistance occurred even if crops other than cotton received few insecticide sprays and were estimated to provide a 95% refuge for cotton (Wu and Guo 2005). This indicates that refuges of non-Bt cotton could be required to significantly delay resistance to Bt cotton. However, key parameters affecting resistance (e.g., dominance of resistance, intensity of selection for resistance) may differ between synthetic insecticides and Bt cotton. Moreover, farmers apply synthetic insecticides at the end of the growing season to minimize bollworm survival on Bt cotton (Wu et al. 2005), which could limit the number of Bt-resistant individuals. Thus, because large non-cotton refuges are available, it was decided that non-Bt cotton refuges are not needed to delay the evolution of *H. armigera* resistance to Bt cotton in China (Wu and Guo 2005). Monitoring of resistance from 1997 to 2006 indicated no increase in resistance to Cry1Ac in China (Wu 2007; but see Li et al. 2007). Wu (2007) proposed that more stringent resistance management measures are needed to delay the evolution of *H. armigera* resistance to Bt cotton in the Yellow River region.

Cotton is grown during the rainy season over a period of 4–5 months in West Africa when weeds sustaining *H. armigera* are rare compared to cotton (Nibouche et al. 2007). Moreover, vegetable crops suitable for *H. armigera* are uncommon during this period and often distant from cotton fields. Nibouche et al. (2003, 2007) used simulation models to assess the area of external refuges of non-Bt cotton required to significantly delay resistance to Bt cotton in *H. armigera*. A period of 20 years before field failure of Bt cotton was deemed a significant delay. Taking into account availability of non-cotton refuges and assuming relatively high survival of *H. armigera* on one- and two-toxin Bt cotton at the end of the growing season, they found that planting less than 25% of the cotton cropping area with one- or two-toxin Bt cotton was required to significantly delay resistance evolution in West Africa. Such a low use of Bt cotton may not be practical for small farmers, especially if field trials demonstrate that Bt cotton can improve farmer income. Local cooperatives in which only a fraction of farmers plant Bt cotton in a given year could be part of a solution to balance resistance risks with improved farmer income. If possible, planting refuges that increase fitness costs near transgenic cotton (see *Fitness costs and incomplete resistance*) could allow farmers to increase their use of Bt cotton. More research is needed to identify host plants that increase fitness costs in *H. armigera* and other key cotton pests in developing countries.

### Effect of seed contamination on management of resistance

Many small farmers could save seed from their current crop to plant the next year's crop (see *Gene flow between transgenic and conventional cotton fields*). This would favor mixing of non-transgenic and transgenic seed. Small farmers could end up unknowingly using seed mixtures under these circumstances. When non-cotton refuges are rare, this would yield a situation similar to planting internal refuges in fields of transgenic cotton and external refuges contaminated with transgenic cotton. When non-cotton refuges are abundant, mixing of transgenic and non-transgenic seed would have less impact on resistance management. While the effect of contamination of external refuges by transgenic cotton has been explored theoretically (Heuberger et al. 2008a), the implication for resistance management of simultaneous contamination of transgenic and non-transgenic cotton is currently under investigation (Heuberger *et al*, in preparation).

Bt toxin production in cotton is expected to be inherited as a dominant trait (see *Gene flow between transgenic and conventional cotton fields*). Thus, a seed mixture arising from recurrent gene flow between one-toxin, true-breeding cotton and non-transgenic cotton would yield plants producing a high concentration of Bt toxin (i.e., plants with one or two copies of the transgene) or no Bt toxin at all. A seed mixture arising from recurrent gene flow between two-toxin, true-breeding cotton and non-transgenic cotton would yield plants producing a high dose of the two toxins, a high dose of either toxin (assuming the transgenes are not located near each other on a chromosome), or no toxin at all. Independent of the type of seed mixtures planted, increased survival of larvae with resistance alleles relative to larvae without resistance alleles in contaminated external refuges could diminish the efficacy of refuges in lowering the heritability of resistance, thereby accelerating resistance evolution (Carrière et al. 2004a, Sisterson et al. 2005, Heuberger et al. 2008a). Moreover, contamination of external refuges could prevent fitness costs from selecting against resistance alleles in refuges, which would also accelerate resistance evolution (Carrière and Tabashnik 2001, Tabashnik et al. 2005).

Redundant killing would be diminished in seed mixtures arising from gene flow between two-toxin cotton and non-transgenic cotton. Thus, in the presence of external refuges, seed mixtures arising from gene flow between two-toxin cotton and non-transgenic cotton would likely accelerate resistance evolution compared to a situation where uncontaminated fields of Bt cotton are planted. The evolution of resistance could also be faster when seed mixtures involving one-toxin cotton are used instead of uncontaminated Bt seed. Larval movement between transgenic and non-transgenic plants could be a critical factor affecting the efficacy of such seed mixtures (Tabashnik 1994b, Carrière et al. 2004a, Fitt et al. 2004, Onstad 2006). In the presence of external refuges, increased survival of heterozygous larvae relative to susceptible larvae in seed mixtures would make resistance less recessive and accelerate the evolution of resistance, compared to a situation where uncontaminated fields of Bt cotton are used. Increased dominance of resistance in seed mixtures could occur if heterozygous larvae are more likely than susceptible larvae to leave transgenic plants and reproduce on non-transgenic plants, or if heterozygous larvae are less likely than susceptible larvae to move from non-transgenic plants and die on transgenic plants.

Increased dominance of resistance is not the only condition that could accelerate the evolution of resistance in seed mixtures. Even a slight increase in survival of both *rs* and *ss* larvae in seed mixtures compared to fields of uncontaminated Bt cotton could hasten the evolution of resistance if larval mortality is density-dependent. In such a situation, a lower density of larvae on non-transgenic plants in seed mixtures than in external refuges could decrease the extent of density-dependent mortality in seed mixtures compared to external refuges. This could increase the relative survival of *rs* larvae in seed mixtures compared to external refuges, which would raise the probability of mating between *rr* and *rs* individuals in seed mixtures and accelerate resistance (Onstad et al. 2002, Crowder and Onstad 2005; see efficacy of their “practical” and “theoretical” high dose strategy). Greater survival of *rs* and *ss* larvae in seed mixtures compared to fields of uncontaminated Bt cotton could occur if a proportion of the *rs* and *ss* larvae that hatch on transgenic plants moved and reproduced successfully on non-transgenic plants. The implications of increased survival of *rs* and *ss* larvae in seed mixtures compared to fields of uncontaminated Bt cotton is currently under investigation (Heuberger *et al*, in preparation). However, changes in survival of *rs* and *ss* larvae in seed mixtures would likely have little effect on resistance evolution when density-dependent mortality of larvae is absent or weak, or when the density of larvae is similar on non-transgenic plants in seed mixtures and in external refuges.

The theoretical possibility that a seed mixture may accelerate the evolution of resistance compared to the use of external refuges has not received strong empirical support. Shelton et al. (2000) and Tang et al. (2001) reported experiments designed to compare durability of seed mixtures and separate refuges, using field and greenhouse tests with diamondback moth, *Plutella xylostella*, and broccoli. However, neither provided direct evidence that the rate of resistance evolution was more rapid in seed mixtures than in transgenic broccoli with external refuges (Carrière et al. 2004a). Tang et al. (2001) reported that *rs* larvae never survived after leaving Bt plants. Thus, the possibility that larval movement in a seed mixture could increase the dominance of resistance was not supported. A recent study of *P. gossypiella* larvae feeding on artificial cotton bolls containing a mixture of seed producing Cry1Ac and seed without Cry1Ac did not support the idea that the dominance of resistance is increased in seed mixtures (Heuberger et al. 2008a). Three types of Bt contamination are found in refuges in Arizona: Bt plants with 100% Bt seeds, Bt plants from hybrid seeds with 70–80% Bt seeds, and outcrossed bolls on non-Bt plants with 5–20% Bt seeds. In this laboratory study, survival of pink bollworm *rr* and *rs* larvae did not differ from survival of *ss* larvae on artificial bolls with a mixture of 20% Bt and 80% non-Bt cotton seeds. On artificial bolls with 70% Bt seeds and 30% non-Bt seeds, or on bolls with 100% Bt seeds, *rr* had higher survival than *ss*, although *rs* and *ss* did not differ. Thus, feeding on a mixture of Bt and non-Bt seeds did not increase the dominance of resistance. Nevertheless, the survival of *rs* and *ss* larvae was equally increased in bolls with a mixture of Bt and non-Bt seeds compared to bolls with 100% Bt seeds. This supports the idea described above that seed mixtures could increase the rate of resistance evolution in the presence of density-dependent larval mortality.

While there is no direct evidence that seed mixtures accelerate resistance evolution, results from behavioral studies indicate that larvae of many pests can avoid Bt toxins, which could increase the dominance of resistance in seed mixtures. Feeding choice studies conducted with the lightbrown apple moth, *Epiphyas postvittana*; the soybean looper, *Pseudoplusia includens*; the beet armyworm, *Spodoptera exigua*; the cabbage looper, *Trichoplusia ni*; and *H. virescens, H. zea*, and *H. armigera* indicate that larvae from these species can avoid feeding on plant tissue with Bt toxins (Li et al. 2006, Heuberger et al. 2008a). In comparison, larvae from relatively few species (*P. gossypiella* and *P. xylostella*) seem to lack the ability to avoid Bt toxins (Heuberger et al. 2008a). Furthermore, newly hatched larvae from several key cotton pests (e.g., *H. virescens, H. zea* and *H. armigera*) clearly have the capability to move between Bt and non-Bt plants in seed mixtures (Parker and Luttrell 1999, Gore et al. 2002, Men et al. 2005). Thus, experiments measuring fitness of newly-hatched *ss, rs*, and *rr* larvae of cotton pests in seed mixtures are needed to evaluate the effect of seed mixtures on resistance to transgenic cotton (Tabashnik 1994b, Carrière et al. 2004a, Fitt et al. 2004).

### Effects of transgenic cotton deployment on resistance to synthetic insecticides

In northern China, cultivation of Bt cotton has substantially reduced the use of synthetic insecticides for the control of *H. armigera* (see *Performance of transgenic cotton in developing countries*). The reduction in selection for insecticide resistance in the five years following the introduction of Bt cotton significantly increased *H. armigera* susceptibility to synthetic insecticides (Wu et al. 2005, Wu 2007). This was likely favored by high availability of refuges provided by crops other than cotton, an increase in refuge area due to less frequent spraying of cotton, and fitness costs associated with resistance (Wu et al. 2005). The higher efficacy of insecticides facilitates the control of *H. armigera* at the end of the growing season and reduces the environmental impacts of cotton cultivation. *H. virescens* showed a similar return to susceptibility in southern Tamaulipas, Mexico, after the introduction of Bt cotton (Terán-Vargas et al. 2005). In the early 1990s, pyrethroids were regularly used for the control of this pest in cotton. By 1995, *H. virescens* had become highly resistant to many types of pyrethroids and control failures occurred. However, high adoption of Bt cotton from 1996 to 2001 coupled with low use of pyrethroids in cotton and other *H. virescens* host crops resulted in significant declines in resistance. By 2001, *H. virescens* susceptibility to pyrethroids had been restored in southern Tamaulipas.

Deployment of Bt cotton does not always result in reversals of resistance to synthetic insecticides. For example, high deployment of Bt cotton in Louisiana did not change the high levels of resistance to pyrethroids in *H. virescens* and *H. zea* (Bagwell et al. 2001). Stability of resistance in this case may be partially explained by a low availability of refuges and marginal declines in selection intensity, as pyrethroids were widely used in other crops attacked by these pests (e.g., maize, sorghum, and soybean). In several regions of Texas, *H. zea* resistance to pyrethroids progressed significantly after the deployment of Bt cotton (Pietrantonio et al. 2007).

### Summary and Outlook

Below we summarize the major facts, patterns, concepts and models outlined in this review and focus of future research needs.

### Development of transgenic cotton cultivars

Transgenic cotton is developed using one of three geneinsertion or transformation techniques, each with advantages and disadvantages. Transformed plants are screened for normal development, good agronomic characteristics, and high levels of transgene expression. Plants with appropriate characteristics are self-fertilized over several generations, during which the stability and heritability of transgene expression is evaluated. Plants that pass this selection process are backcrossed with a commercial cultivar several times. Selfing is used to finally produce an agronomically viable homozygous transgenic cultivar. Farmers may use such transgenic cultivar, or use a hybrid cultivar resulting from a cross between the homozygous transgenic cultivar and a non-transgenic cultivar. To ensure high performance, transgenic cultivars produced by backcrossing or hybridization must be rigorously field tested where they will be grown commercially.

### Efficacy of transgenic cotton against arthropod pests

Most transgenic cotton cultivars contain genes from the bacterium *B. thuringiensis* that confer insecticidal properties to the plant. However, two cultivars produce insecticidal proteins derived from plants other than cotton. Most transgenic cotton cultivars target bollworm or budworm lepidopteran pests, which are the most economically important pests of cotton throughout the world. Two transgenic cultivars in development will specifically target aphids and armyworms. Commercialized cultivars vary in their control of bollworms and budworms; control varies with experimental conditions from low to high. Commercialized cultivars may provide moderate protection against Lepidoptera other than bollworms and budworms and no protection against non-lepidopteran pests.

### Performance of transgenic cotton in developing countries

For growers in developed and developing countries, transgenic cotton has helped to increase yield, decrease insecticide use, or both. Growers of transgenic cotton in China significantly reduced their insecticide use but saw only minor yield increases, likely because key cotton pests have been relatively well controlled by insecticides. In India where only Bt hybrids are available, farmers significantly increased their yield and decreased insecticide use by adopting Bt cotton. However, low performance of Bt hybrids was reported in some regions with poor growing conditions. Such low performance was likely due to a combination of factors, including the use of Bt hybrids poorly adapted to local growing conditions, use of uncertified or saved seed, high regional use of insecticides, and years of low bollworm infestations. South African smallholder farmers from the Makhathini Flats increased their yields and lowered their insecticide use with Bt cotton. However, some studies conducted in South Africa may have primarily investigated farmers with higher than average access to credit and technical support. Thus, studies examining the performance of transgenic cotton across a wider range of growing practices could be useful to more comprehensively assess the performance of transgenic cotton in Africa. The lower use of insecticides in Bt than non-Bt cotton sometimes increased or decreased problems caused by pests that are not killed by Bt cotton. Problems increased in Bt cotton when insecticides were required to control pests not affected by Bt cotton, while problems decreased when the reduction in insecticide use benefited natural enemies and improved biocontrol.

### Factors affecting transgene expression

Changes in transgene expression leading to a reduction in the concentration of Bt toxins can result in increased insect damage and lower performance of transgenic cotton cultivars. Toxin production can vary among cultivars, over the growing season, and between plant parts. Such variation can be attributed to characteristics of the plant's genome (including the transgene), physiology, and phenology. Environmental factors such as soil nitrogen levels and temperature can result in changes in the concentration or toxicity of Bt toxins. Bt cotton has higher nitrogen content than non-Bt cotton, suggesting that Bt cotton may have greater nitrogen uptake and metabolism than conventional cultivars. Performance studies conducted in China, India and South Africa indicate that such differences in nitrogen requirements may have little effect on yield. However, more research is needed to assess whether the relative performance of Bt cotton will be maintained across the range of soil nitrogen content typically encountered in developing countries. High temperatures may also reduce toxin production by stressing the plants and altering their nitrogen metabolism, although there is currently no evidence that high temperatures negatively affect yield.

### Gene flow between transgenic and conventional cotton

Unwanted gene flow between transgenic and conventional cotton could be problematic for insect resistance management strategies, organic cotton production, and maintenance of the intellectual property rights of biotechnology firms. Moreover, gene flow from transgenic cotton to wild cotton species could endanger the genetic integrity of native species. Buffers of 60m or less of non-transgenic cotton can be used to limit gene flow from transgenic cotton fields. Cotton growers relying on buffers should make sure that buffers are only planted with certified non-transgenic seed. Fields with abundant pollinating insects may require larger buffers than fields with limited insect activity. Growers should exercise caution when planting transgenic cotton in areas with genetically compatible wild cotton species and are advised to use large buffers or avoid using transgenic cotton in those regions. In regions where farm-saved seed is used, precautions should be taken to protect conventional cotton cultivars, as farm-saved seed is particularly vulnerable to gene flow from transgenic varieties. Similarly, providing affordable, certified seed of transgenic cotton to farmers could help preserving integrity of transgenic cultivars. This would contribute to preserving insecticidal properties of the transgenic cultivars and to the sustainable management of pest resistance.

### Non-target effects of transgenic cotton

Insecticide use can be much lower in Bt cotton than in non-Bt cotton. Thus, assessments of non-target effects of Bt cotton should consider patterns of insecticide use in Bt and non-Bt cotton. The abundance of non-target insects often declined moderately but significantly in unsprayed Bt cotton compared to unsprayed non-Bt cotton, presumably due to a reduction in the abundance of lepidopteran insects that are important resources for non-target arthropods. The reductions in predator abundance in unsprayed Bt cotton compared to unsprayed non-Bt cotton did not affect predation rates, suggesting that predation rates in cotton can be sustained despite declines in abundance of certain predators. Lower insecticide use in Bt cotton than in non-Bt cotton often benefited non-target organisms in Bt cotton and improved biocontrol in some cases. However, decreased abundance of parasitoids specializing on lepidopteran pests occurred in Bt cotton in parallel with decreased abundance of lepidopteran hosts. Overall, few adverse effects on non-target arthropods have been observed for cotton producing the Bt toxin Cry1Ac. More data are needed to thoroughly assess impacts of Cry1Ac cotton on soil organisms, as well as impacts of transgenic cultivars producing toxins other than Cry1Ac.

### Management of pest resistance to transgenic cotton

The refuge strategy is widely used to delay pest resistance to transgenic cotton. This strategy is most effective when resistance is recessive and sufficient refuges of non-transgenic host plants are available. Transgenic cotton that kills < 99% of susceptible insects may need large refuges to significantly delay resistance. Fitness costs and incomplete resistance can be exploited to delay resistance evolution. Research is needed to identify plants or natural enemies that increase fitness costs in *H. armigera* and other key cotton pests in developing countries. After more than a decade of use of transgenic crops, one key pest of cotton, *H. zea*, has evolved resistance in the field to Bt cotton producing Cry1Ac. Furthermore, a second key pest, *H. amigera*, may be in the process of doing so. Two key pests of corn, *B. fusca* and *S. frugiperda*, have evolved resistance in the field to Bt corn producing Cry1Ab and Cry1F, respectively. Because factors influencing resistance evolution often change across regions, region-specific evaluation of resistance risks and implementation of resistance management is required to sustain the effectiveness of transgenic cotton for pest control. While the refuge strategy has so far been implemented in countries where seed purity is high, many small farmers in developing countries where transgenic cotton will be introduced could plant their cotton fields with farm-saved seed. In such cases, small farmers may end up unknowingly planting seed mixtures of transgenic and non-transgenic cotton. The possibility that seed mixtures of non-transgenic and transgenic cotton may accelerate the evolution of resistance needs critical investigation. Experiments assessing fitness of larvae with and without resistance alleles in seed mixtures are needed to rigorously evaluate this possibility. In some cases, the deployment of Bt cotton associated with lower use of insecticidal sprays has reversed pest resistance to synthetic insecticides, thereby increasing options for controlling key cotton pests.

## Figures and Tables

**Appendix A.**
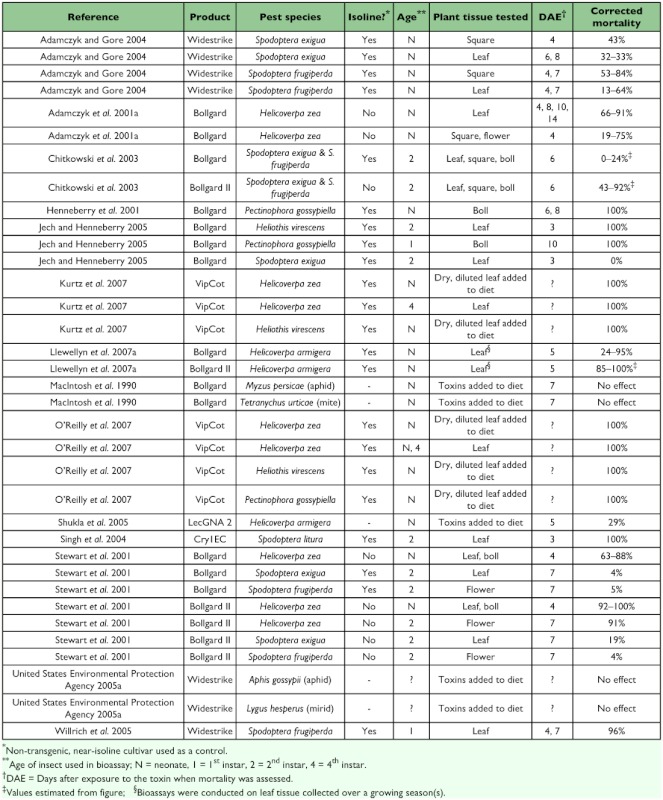
Summary of data used to calculate mortality caused by transgenic cotton in Table 2.

**Appendix B.**
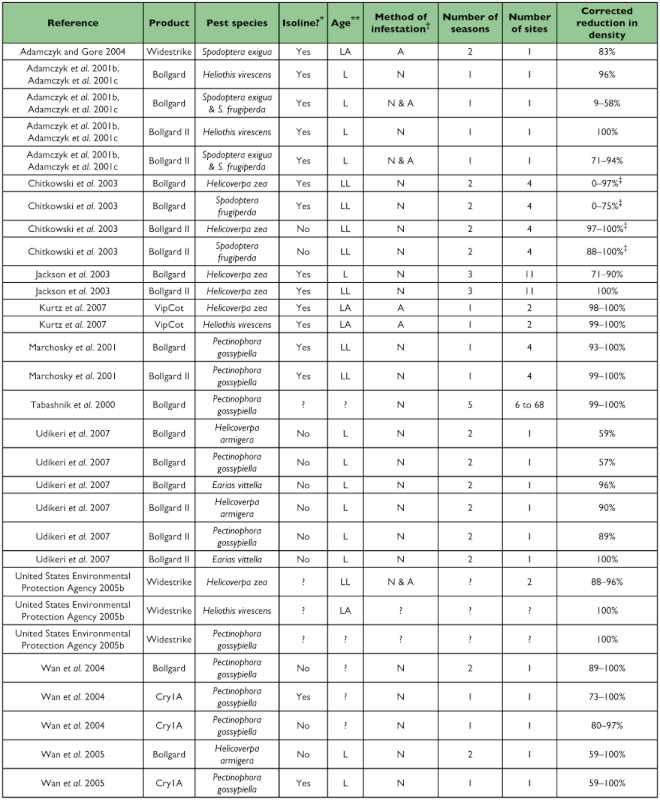
Summary of data used to calculate reduction in population density in fields of transgenic cotton compared to nontransgenic cotton in Table 3.

## Glossary Terms

Additive fitness costs: If r is a resistance allele and s an allele for susceptibility costs are additive when fitness of rs is halfway between fitness of ss and rr on non-transgenic plants.
Allele: One of several forms of a gene.
Condensed tannins: Anti-herbivory protein-binding molecules found in plants.
Constitutive promoter: Region of DNA regulating gene transcription in all parts of the plant at all times.
Cross-resistance: When a genetically-based decrease in susceptibility to one toxin also decreases susceptibility to other toxins.
Dominance: The extent to which heterozygous or hemizygous individuals have the same phenotype as homozygous individuals.
Dominant resistance: If r is a resistance allele and s an allele for susceptibility resistance is dominant when resistance of rs is equal to resistance of rr.
Fitness: The average contribution of one genotype to the next generation compared to another genotype.
Fitness cost: When fitness on non-transgenic plants is lower for individuals with resistance alleles than for individuals without resistance alleles.
Gene silencing: Occurs when multiple transgene loci reduce transgene expression.
Genotype: The set of genes possessed by an individual organism. Genotype may be used to describe a subset of genes such as genes involved in resistance to a toxin.
Heritability: A measure of the resemblance between parents and offspring due to the effect of genes.
Hemizygous: Having only one allele at a locus as in hybrid genetically modified organisms at the locus of an insertion event.
Heterosis: When hybrid plants are more vigorous than either of their parental cultivars.
Heterozygous: Having different alleles at a locus. **Homozygous:** Having the same allele at a locus.
Hybrid counterparts: Hybrids that share the same parental genome except for the presence of a transgene(s) the hybrid equivalent of isogenic cultivars.
Hybrid transgenic cultivar: A cultivar obtained by crossing a transgenic cultivar and a non-transgenic cultivar. Such a plant is hemizygous for the transgene and generally possesses a mixture of the characteristics of parental plants.
Inducible promoter: Region of DNA regulating gene transcription during certain developmental stages or in response to environmental stimuli.
Isogenic cultivars: Cultivars that share the same genetic background or genotype except for possession of one or more transgenes. Isogenic transgenic and non-transgenic cultivars are useful for determining the phenotypic effects of transgenes (e.g. effect on yield).
Locus: A site on a chromosome occupied by a specific gene.
mRNA: Molecule transcribed from DNA that carries coding information to the sites of protein synthesis. Also called “messenger RNA”.
Non-recessive fitness costs: If r is a resistance allele and s an allele for susceptibility costs are non-recessive when fitness of rs is less than fitness of ss on non-transgenic plants.
Phenotype: Properties of an organism as described by behavioral biochemical life history morphological or physiological characters.
Plasmid: Circular piece of DNA.
Promoter: Region of DNA regulating gene transcription that determines where when and how much of a gene is expressed.
Protease: Enzyme involved in protein digestion.
Pyramid strategy: The strategy of using cultivars that produce two distinct toxins to delay resistance.
Recessive fitness costs: If r is a resistance allele and s an allele for susceptibility costs are recessive when fitness of ss is equal to fitness of rs on non-transgenic plants.
Recessive resistance: If r is a resistance allele and s an allele for susceptibility resistance is recessive when resistance of rs is equal to resistance of ss.
Tissue-specific promoter: Region of DNA regulating gene transcription only in specific plant parts.
Transcription factor: Molecule that binds to a promoter and other DNA sequences and helps to stabilize the transcription of a gene.
True-breeding transgenic cultivar: Gultivar with two identical transgenes at a locus. Also known as a homozygous transgenic cultivar.
Volunteer plants: Plants that grow from seed left by the previous crop.
